# Preclinical profiling of antibody drug conjugates targeting oncofetal chondroitin sulfate

**DOI:** 10.1038/s41419-026-08420-x

**Published:** 2026-01-24

**Authors:** Ann Skafte, Elena Ethel Vidal-Calvo, Swati Choudhary, Joana Mujollari, Robert Dagil, Anne Martin-Salazar, Htoo Zarni Oo, Lara Duvnjak, Thor G. Theander, Mads Daugaard, Tobias Gustavsson, Ali Salanti

**Affiliations:** 1 Centre for Translational Medicine and Parasitology, Department of Immunology and Microbiology, Faculty of Health and Medical Sciences, University of Copenhagen, Copenhagen University Hospital, Copenhagen, Denmark; 2 VAR2 Pharmaceuticals ApS, Frederiksberg, Denmark; 3 Vancouver Prostate Centre, Vancouver Coastal Health Research Institutes, Vancouver, BC Canada

**Keywords:** Drug development, Targeted therapies

## Abstract

Antibody-drug conjugates (ADC) offer a targeted cancer treatment approach by delivering cytotoxic payloads directly to tumor cells. However, resistance mechanisms, poor tumor penetration, and off-target toxicity often limit clinical efficacy. Vartumab targets oncofetal chondroitin sulfate (ofCS), a pan-cancer target present on tumor cells and in the malignant stroma, with low expression in normal tissues. As part of transitioning Vartumab to clinical evaluation, two linker-payloads known to mediate bystander effects, valine-citrulline (vc)—monomethyl auristatin E (MMAE) and glycine-glycine-phenylalanine-glycine (ggfg)—Deruxtecan (DXd), were investigated for design of Vartumab ADCs. We show that the ADCs maintain specificity to ofCS proteoglycans, cancer cells, and tissue biopsies, exhibiting specific binding to a wide range of malignant and metastatic tissues. Biodistribution assessment of Vartumab ADCs in mice shows strong and specific tumor uptake, with minimal accumulation in other organs. Both ADCs induced bystander killing of antigen-negative cells in the presence of antigen-positive cells and displayed potent anti-tumor activities in a cell-derived xenograft melanoma model. Furthermore, we show that Vartumab conjugates with bystander-capable linker-payloads exhibit greater in vivo potency compared to those with payloads lacking significant bystander effect. Finally, toxicity assessment in rats indicates that the ADC-MMAE is well-tolerated upon repeated doses, with similar dose-limiting toxicities as reported for clinically approved MMAE-conjugated ADCs. Our data support further clinical development of Vartumab-based ADCs.

## Background

Cancer is a major global health problem affecting millions of people each year. By 2050, the annual number of new cases is predicted to increase by 77% compared to 2022, emphasizing the critical need for new treatment options [1]. While surgery, chemotherapy, and radiotherapy often remain the first-line therapeutic choice, the emergence of monoclonal antibodies and the development of targeted cancer therapies, such as immunotherapies and antibody-drug conjugates (ADC), have paved the way for precision oncology. ADCs combine a tumor-specific antibody with a toxic payload, aiming to selectively target and kill tumor cells while minimizing the adverse effects commonly associated with the administration of systemic chemotherapy [2]. By the end of 2022, 431 ADCs had entered clinical trials, but only 14 ADCs had received FDA approval, highlighting the challenges in developing effective ADCs [3]. Failures in development have been attributed to various factors, such as heterogeneous target expression or down-regulation of the target antigen, different resistance mechanisms [4], including resistance to the payload [5], ineffective therapy delivery to the tumor due to a dense extracellular matrix (ECM), poorly vascularized tumor [6], or on-target off-tumor toxicity through antibody binding to healthy tissue causing severe side-effects [4, 7, 8].

ADCs exert their effect by internalization into the effector cell upon binding cell surface antigens and/or through bystander effects [9–11]. In the latter, after killing the target cell, the released cytotoxic payload, when non-polar, diffuses into and kills neighboring cells irrespective of antigen expression level. Bystander effects can also be exerted by the release of the toxin in the tumor stroma, allowing killing of both cancer cells and supportive stromal cells [12, 13]. For this to happen, the ADC must be designed with a cleavable linker and a hydrophobic and membrane-permeable payload [14]. Of the FDA-approved ADCs used in clinics for solid tumors, some have shown preclinical evidence of bystander effect through killing of antigen-negative (Ag-) cells using MMAE and DXd payloads, like trastuzumab deruxtecan and enfortumab vedotin [13, 14]. Both payloads are membrane permeable and exert their effect by either blocking microtubule polymerization, causing cell arrest (e.g., MMAE), or interfering with DNA replication (e.g., by inhibiting topoisomerase 1 and locking the DNA strand in a broken state for DXd), thereby inducing apoptotic cell death [11, 15–17]. The ggfg-DXd linker payload, as used in trastuzumab deruxtecan, has been shown recently to outperform trastuzumab emtansine, designed with a non-cleavable linker, in a phase III trial for HER2-positive metastatic breast cancer [18]. This superior efficacy is speculated to be attributed to the extracellular linker cleavage and bystander killing of nearby cancer cells regardless of antigen expression levels [18, 19], highlighting the importance of evaluating both linker and payload in next-generation ADCs.

Chondroitin sulfate (CS) is a group of glycosaminoglycans, which are carbohydrate chains covalently attached to various proteins in the cell membrane of cancer cells and tumor-associated stromal cells, but also excreted into the surrounding ECM [20]. While CS is present throughout the body, distinct CS variants play a key role in tumor progression and tumor immune escape in cancer, similarities shared with developmental processes in embryonic tissues [21–25]. In placental malaria pathogenesis, the *Plasmodium falciparum* parasite-encoded protein, VAR2CSA, naturally interacts with a specific CS subtype found exclusively on syncytial trophoblasts [26]. We demonstrated that recombinant VAR2CSA also binds the vast majority of solid tumor biopsies and circulating tumor cells, mediated through this distinct type of CS termed oncofetal chondroitin sulfate (ofCS) [20, 27]. In cancer tissue, ofCS has been characterized as unusually long and highly 6-O and 4-O sulfated CS chains [28]. Through antibody phage-display panning on ofCS, we identified an antibody fragment with high affinity and specificity to ofCS [29]. The antibody fragment, termed Vartumab, bound to the vast majority of cancer biopsies irrespective of their origin, with minimal binding to most healthy tissues [29]. Interestingly, we found that ofCS appears both on the malignant cells, in the ECM and on the cancer associated fibroblasts, while being absent on non-tumor associated fibroblasts and healthy adjacent tissue. Targeting the tumor with a Vartumab ADC would thus serve the dual function of targeting the cancer cells and the tumor supportive stromal cells, potentially offering a more complete and lasting tumor regression. Supporting this notion, Vartumab functionalized with an MMAE toxin demonstrated strong anti-tumor efficacy in a range of animal models, including cell-derived and patient-derived xenografts [29]. Vartumab is currently being tested in a PET/CT phase 0 imaging trial in 16 patients to determine biodistribution in cancers of both epithelial and mesenchymal origin (ClinicalTrials.gov ID: NCT06645808).

Here, we present the assessment of two linker-payloads conjugated to the anti-ofCS antibody fragment Vartumab: the microtubule inhibitor valine-citrulline (vc)-MMAE and the topoisomerase 1 inhibitor glycine-glycine-phenylalanine-glycine (ggfg)-DXd. We evaluate the Vartumab ADCs based on in vitro binding, anti-tumor effect, bio-distribution in animal models, and showcase that bystander effect is needed to drive complete anti-tumor efficacy in vitro and in vivo by comparing with a linker payload having reduced membrane permeability. The safety profile of the vc-MMAE ADC was assessed in a repeat dose tolerability study in rats.

## Materials and methods

### Ethical permits

Animal studies were approved by the Animal Experiments Inspectorate (P23-433). Ethical approvals were obtained from the Animal Experiments Inspectorate, Danmark. All methods were performed in accordance with the national guidelines and regulations.

### Protein production

Synthetic DNA encoding the gene for each construct was purchased from GeneArt (ThermoFisher). Control Protein (ABDwt-SpyC-2Cys) and Vartumab (C9scFv-ABDwt-1Cys) had NotI and NcoI restriction enzyme sites for cloning, a C-terminal 6xHistidine tag for purification, and when indicated a C-terminal V5 tag. All genes were ligated into a pET28 vector (Novagen), and plasmids were sanger sequence verified (Eurofins). Plasmid with the control construct was transformed into *Escherichia coli* (*E. coli*) BL21 DE3 (New England Biolabs, #C2527H) while plasmids containing Vartumab and chABC (Uniprot C7S340) were transformed into *E. coli* Shuffle T7 express (New England Biolabs, # C3029J) for subsequent protein expression. Cells were grown in 2xYT media at 37 °C until early exponential phase, upon which temperature was decreased to 20 °C followed by induction with 0.1 M isopropyl β-D-1-thiogalactopyranoside (IPTG). After 16 h, the cells were harvested by centrifugation. Cells were lysed in phosphate buffer, and the soluble fraction was purified on an IMAC column (HisTrap HP, Cytiva #17524802). Following IMAC purification, the control protein was purified using an anion exchange column HiTrap Q HP (HP, Cytiva #17115301) while Vartumab and chABC enzyme were further purified using a size exclusion column Sephacryl S-300 HR equilibrated with phosphate buffered saline (PBS) pH 7.4 (Cytiva #17059910).

### Cell lines

A list of cell lines used can be found in Supplementary Table 1 with origin, disease, and accession number (Cellosaurus ID). A375 WT and B4GALT7 KO were kindly gifted by Charlotte Spliid from University of California, San Diego, USA. DU145 and UM-UC.13 were kindly gifted by Mads Daugaard at Vancouver Prostate Centre, Canada. U-138 MG was kindly provided by Janine Erler and Lara Perryman at Biotech Research & Innovation Centre (Copenhagen) [30], and NCI-H1975 was purchased from ATCC. Culture media and growth conditions for cell lines and passaging were done according to ATCC guidelines. On the day of experiments, cells were detached using Accutase (Gibco^TM^, #A11105-01) to maintain ofCS surface expression. Cell cultures were tested for mycoplasma contamination regularly, and all experiments were performed with mycoplasma-free cells. Cell lines were not authenticated, as genetic background was not essential for drawing conclusions.

### Conjugation

Maleimidocaproyl-vc-PABC-MMAE (MedChem Express, #HY-15575) and Maleimidocaproyl-ggfg-DXd (Deruxtecan) (Broadpharm, #BP-29531) were both dissolved to a concentration of 10 mM in DMSO prior to conjugation. For the control construct, the protein was reduced using 3 molar excess (ME) of tris (2-carboxyethyl) phosphine (TCEP) for 90 min at 37 °C, followed by conjugation for 60 min at room temperature (RT) using 2ME of either linker-payload. For Vartumab, the antibody was directly incubated with 2.5ME linker-payload for 60 min at RT. Vartumab was also conjugated with Maleimidocaproyl-vc-MMAF (MedChem Express, #HY-112786) using the same procedure. To remove unreacted toxin, all conjugated proteins were purified using Zeba Spin Columns (Thermo Scientific, #89878) using manufacturer guidelines and stored at −80 °C. Resulting conjugates were termed ADC for Vartumabs, and control drug conjugate (CDC) for controls. For cathepsin B and L digestion of ADCs and CDCs, enzyme was incubated with drug conjugate at a ratio of 1:1000 enzyme:drug conjugate for Cathepsin B (R&D Systems, #953-CY) and 1:40,000 for Cathepsin L (R&D Systems, #952-CY). Assay buffers are as defined by the manufacturer. Drug conjugates were incubated at RT for 24 h before neutralization and subsequent analysis on SDS-PAGE.

### Intact mass spectrometry

For MS analysis, unconjugated protein, along with ADCs and CDCs were desalted using ZipTip C18 (Millipore) pipette tips. Briefly, proteins were acidified using 1% trifluoroacetic acid (TFA) and loaded onto the ZipTip resin and washed with 0.1% TFA before spotting on MTP 384 ground steel target plate using HCCA in 0.1% TFA as matrix. From the resulting mass spectra obtained from a MALDI-TOF Autoflex (Bruker) instrument, the drug-antibody ratio (DAR) was calculated as the mass difference between unconjugated protein and conjugated peaks, divided by the mass of the linker-payload (vc-MMAE = 1316.63 Da, ggfg-DXd = 1034.1 Da).

### SDS-PAGE

One microgram of ADC mixed with 1x loading buffer containing dye and sodium dodecyl sulfate (SDS) was run with dithiothreitol (DTT) and without. After heating the samples at 95 °C for 2 min, they were loaded onto NuPAGE 4–12% Bis-Tris (Thermo Scientific, #NP0322 & #NP0323) gels together with a commercially available protein ladder (Page Ruler Plus Pre-Stained Ladder, Thermo Fischer). The gels were run for either 1 h at 180 V in 3-(N-morpholino) propanesulfonic acid (MOPS) buffer or 1 h and 15 min at 150 V in 2-(N-morpholino) ethanesulfonic acid (MES) buffer and stained in Instant Blue Coomassie Protein stain (Kementec, #Inst-1L-181) for visualization of the protein bands.

### Western blot

Samples run on SDS-PAGE gel were transferred using the iBlot three Western Blot System (Thermo Fisher) following manufacturer’s instructions and kits, using the Broad Range setting. Transfer to the membrane was checked by incubation with Ponceau staining, which was washed off before antibody incubation. The membrane was put on the iBind Flex Western Device (Thermo Fisher) for incubation with V5 Tag Monoclonal Antibody-HRP (Invitrogen, #R961-25, 1:5000) or His Antibody-HRP (C-term) (Miltenyi Biotec, #130-092-783, 1:3000) and washed, following manufacturer’s guidelines. Development was performed using West Atto Super Detection kit (Thermo Scientific, #A38555), and resulting signals were read on chemiluminescence settings on Biorad ChemiDoc.

### Stability studies

For buffer stability study, ADCs were diluted to 1 mg/mL in either PBS pH 7.4 or PBS + 5mM L-Cysteine pH 7.4 and incubated at RT for a week, taking samples at 24 h intervals. Samples were analyzed on SDS-PAGE using methods described above.

For plasma stability study, ADCs were incubated in human plasma at a concentration of 20 µg/mL, at 37 °C for a week, taking samples at 24 h intervals. Samples were analyzed using Western Blot.

### ELISA

96-well plates (Falcon, #351172) were coated with 3 µg/mL decorin (Sigma Aldrich, #D8428) overnight (ON) at 4 °C. Plates were blocked with PBS + 0.05% (v/v) Tween 20 (PBST) containing 5% (w/v) skim milk powder (Blocking Buffer), for 1 h at 37 °C, 110 rpm. Unconjugated protein, ADCs, and CDCs were pre-incubated with 5ME of human serum albumin (Sigma Aldrich, #A7736) for 15 min before serial dilutions in concentrations between 1200 nM and 2.3 nM and incubated for 30 min at 37 °C, 110 rpm. All samples were run in technical triplicates. Following washing three times with PBST, the plates were incubated with V5 Tag Monoclonal Antibody-HRP (Invitrogen, #R961-25, 1:5000) or His Antibody-HRP (C-term) (Miltenyi Biotec, #130-092-783, 1:3000) diluted in blocking buffer for 30 min at 37 °C, 110 rpm. Plates were washed 3x in PBST, developed for 7–8 min using TMB Plus2 (Kementec, #4395A), and the reaction was quenched with 0.2 M H_2_SO_4_. The absorbance at 450 nm was measured on an ELISA reader (Biosan), and results were analyzed using GraphPad Prism, and curves were edited in Illustrator (Adobe).

### Attana biosensor

To determine the binding kinetics, an Attana A200 quartz crystal microbalance (QCM) biosensor (Attana AB) instrument was used. Two LNB carboxyl chips were equilibrated at a flow rate of 10 μl/min in HBS-T coupling buffer (10 mM HEPES, 150 mM NaCl, 0.005% Triton-x100, pH 7.4). Next, chip surfaces were activated using a 1:1 mix of 0.1M N-hydroxysulfosuccinimide (S-NHS) and 0.4 M 1-ethyl-3-(3-dimethylaminopropyl) carbodiimide hydrochloride (EDC) and conjugated through primary amines with 50 μg/ml Streptavidin, following the manufacturer’s instructions. Blocking of the chip surface was done using 1 M ethanolamine. Next, 50 μg/ml placental-purified biotinylated ofCS diluted into 1xPBS (pH 7.4) was injected over the main chip, a frequency shift of ~90 Hz, indicated a mass change after CS binding. The reference chip was kept uncoated to assess the background binding. After equilibration in running buffer (1xPBS, pH 7.4), a 2-fold dilution series of each analyte from 1200 nM to 37.5 nM was injected at a flow rate of 20 μl/min to assess the protein binding to ofCS. The chips were regenerated using 0.01 M NaOH. The binding kinetics (*k*_on_, *k*_off_) were fitted using a 1:2 binding model in TraceDrawer software (Ridgeview Instruments AB).

### Flow cytometry

Adherent cells were detached, counted, diluted in PBS with 2% Fetal Bovine Serum (Gibco, #A5256701) (PBS2), and added to a 96-well round bottom plate (Corning, #3799) at a seeding density of 150,000 cells per well. As control, cells were enzymatically treated using chondroitinase ABC at 0.5 mg/1 × 10^6^ cells for 30 min at 37 °C. Unconjugated protein, ADCs, and CDCs were diluted in PBS2 (3-fold dilution), ranging between 1200 nM and 2.3 nM. The plate was spun to remove PBS2 and resuspended in the protein dilutions for 30 min. All samples were run in technical triplicate. The cells were washed three times with PBS2 before adding V5 Tag Monoclonal Antibody-FITC (Invitrogen, #R963-25, 1:500) or penta-His-A488 (Qiagen, #35310, 1:200). The cells were incubated for 30 min before washing three times with PBS2. The cells were resuspended in 200 µl PBS2 and read on Cytoflex (Beckman Coulter). All incubations were done at 4 °C, shaking at 125 rpm. Data were analysed using FlowJo^TM^ (Becton Dickinson) software. Cell populations were selected by the Side and Forward scatter plot (SSC/FSC), and single cells selected by the FSC-H/FSC-A plot. Geometric Mean Fluorescence Intensity (MFI) was obtained from the fluorescence channel, and relative MFI was calculated by normalizing MFI to the antibody control. All curves were subsequently edited in Illustrator (Adobe).

### Immunofluorescence staining of paraffin-embedded fixed tissues

Multi-organ tissue micro-arrays (BCN1021) were purchased from Biomax. Mouse tissues were prepared and fixed as previously described [29]. All tissues were processed and stained as in [29] with 25 nM ADC-MMAE, ADC-DXd, or Vartumab, and detected with Anti-V5-Alexa647 (Invitrogen, #451098) diluted 1:400 in PBS+0.25% BSA. Imaging was performed on a Zeiss Axio Z1 automated slide scanner (×20 magnification, 0.8 NA objective), and image analysis on the Zen blue software (Zeiss).

### Cytotoxicity

Cells were detached, counted, and diluted in the cell lines’ respective media with 5% Fetal Bovine Serum and 1% Penicillin/Streptomycin (Merck, #P0781), at a density of 3000 cells/well. 24 h after seeding cells, the media was removed from the wells. ADCs were thawed, spun down at 16000 g for 5 min, and diluted in the cell media at concentrations ranging from 2000 nM to 7.8 nM using 2-fold serial dilutions, and 100 µl ADC dilutions were added to the wells. All dilutions were run in technical triplicates. The plates were left to incubate for 72 h at 37 °C in a 5% CO_2_ atmosphere. After incubation, the plates were developed using the Cell Proliferation Kit II (XTT) (Roche, #11465015001). Plates were read using a multi-well spectrophotometer set at 450 nm and 632 nm. Sigmoidal curves for the cell viability vs. log concentration of the ADC were plotted using GraphPad Prism, and the IC_50_ values were calculated using four parameter logistic non-linear regression fit model. All curves were subsequently edited in Illustrator (Adobe).

### Co-culture assays

Melanoma A375 *B4GALT7* KO cells were detached, spun down at 380 g for 5 min, resuspended in serum-free media containing CellTracker^TM^ Green CMFDA (Invitrogen, #C7025) and incubated at 37 °C for 30 min. Following incubation, cells were spun down at 380 g for 5 min, resuspended in supplemented media, and let to rest for 30 min at 37 °C before seeding a 24-well plate (Corning, #3524). Melanoma A375 WT cells were detached and resuspended with the A375 *B4GALT7* KO before seeding into a 24-well plate (Corning, #3524) at fixed density of 100,000 A375 *B4GALT7* KO/well, while varying the density of A375 WT cells from 0 to 100,000/well. The plate was then incubated overnight at 37 °C. The following day, ADCs were prepared in cell media, added to the plate, and incubated in the dark for 72 h at 37 °C. ADC concentrations were >IC_80_ for WT cells and <IC_50_ for *B4GALT7* KO cells (equivalent to ADC-MMAE = 100 nM, ADC-DXd = 600 nM, ADC-MMAF = 50 nM) and added to the plate. ADC-MMAF was used as a control, as it is known for being unable to induce bystander effect. All samples were run in technical triplicate. After 72 h, ADC solutions were removed from the plate, wells were washed 3x with PBS, and cells detached using Accutase (Gibco^TM^, #A11105-01) for 5 min. Cells were spun down, resuspended in PBS2, transferred to a 96-well round-bottom plate (Corning, #3799), incubated with 0.5 µg/ml 4′,6-diamidino-2-phenylindole (DAPI, Invitrogen, #D1306) for 15 min at RT, before washing 3x with PBS2. Plates were read on LSRFortessa 5 (BD Biosciences). Data were analysed using FlowJo^TM^ (Becton Dickinson) software, and curves were edited in Illustrator (Adobe).

### Oncofetal-chondroitin sulfate staining of A375 cells

Melanoma A375 *B4GALT7* cells were labeled with CellTracker^TM^ Green CMFDA (Invitrogen, #C7025) following the methods outlined in the previous section. A total of 80.000 labeled cells were mixed with 40.000 A375 WT cells in 500 µl supplemented DMEM medium, seeded onto a 10 mm coverslip placed in a 24-well plate, and incubated overnight at 37 °C, in a 5% CO_2_ atmosphere. The next day, cell media was removed, cells were carefully washed 2x with ice-cold DMEM medium, followed by 2x with cold PBS2, before fixation with 4% paraformaldehyde for 10 min at room temperature (RT). Cover slips were washed 2x for 5 min with PBS and transferred to a humidified staining chamber. 25 nM C9-scFv^2^ diluted in PBS2 was added to the coverslip and incubated for 1 h, RT. Following 3 × 5 min washes with PBS, anti-V5-Alexa647 (Invitrogen, #451098) secondary antibody was added and incubated 1 h, RT. Following two washes with PBS, counter-staining with DAPI (5 μg/ml, Invitrogen, #D1306) was performed in PBS for 5 min, RT. Coverslips were washed again 2 × 5 min in PBS and 2 × 5 min in Ultra-pure-water, dried for 30 min, and mounted with an aqueous mounting media (Dako, #33025). Confocal imaging was performed on a Zeiss LSM 900 microscope (63x magnification, 1.4 NA), and images were analyzed in Zen blue software (Zeiss).

### Cell-derived xenograft mice models

Female BALB/cAnNRJ-Foxn1^nu/nu^ mice at age 6–8 weeks were purchased from Janvier. Experiments and housing of the animals were at the Department for Experimental Medicine at the University of Copenhagen. The mice were maintained in a 12 h light cycle with a humidity level of 40–60% at 20 °C, handled in accordance with FELASA Rodent Health Surveillance program, and were provided with enrichment opportunities. All experiments were planned considering the three R’s: replacement, reduction, and refinement. The number of mice used in each study was chosen according to previous experiments, ensuring sufficient sample sizes which would give meaningful differences between groups.

Cancer cells were thawed and cultured for a minimum of three passages before injection. Cell suspensions were prepared in PBS and injected subcutaneously on the right flank of the animal using a 25 G needle as follows: A375 WT (1 × 10^6^cells/mouse), NCI-H1975 (4 × 10^6^ cells/mouse). Tumor size was measured with a caliper, and tumor volume was calculated as 0.5236*length*width^2^, assuming height is identical to width. Weight was measured at the same time as tumor volume.

### In vivo efficacy

Mice were randomized manually using tumor volumes and randomly placed in groups of an average tumor size of 100–150 mm^3^ across treatment groups. Mice were excluded from the study if tumor volume was above 175 mm^3^ or below 90 mm^3^ before randomization. Treatment dosing was based on literature [31] and adjusted for the size of the ADCs (kDa), at either 1.6 nmol toxin or 3.2 nmol toxin, corresponding to 3 mg/kg and 6 mg/kg for Vartumab groups (ADC-MMAE DAR = 0.9, ADC-DXd DAR = 0.8). CDC groups (CDC-MMAE DAR = 1.9, CDC-DXd DAR = 1.8) were dosed with equimolar amounts of toxin, corresponding to 0.8 mg/kg and 1.6 mg/kg. For the bystander efficacy study in mice, ADC-MMAE and ADC-MMAF were dosed 2.1 nmol toxin corresponding to 3.7 mg/kg. For the tumor recurrence in mice, ADC-MMAE was dosed with equimolar amounts of toxin to the bystander efficacy study at 2.1 nmol toxin, corresponding to 6.6 mg/kg. Two treatments were administered intravenously in the tail vein and given 5 days apart for all efficacy studies. Tumor volumes and mice weight were followed 2–3 times per week, and mice were euthanized when they reached the humane endpoint (tumor volume exceeding 1000 mm^3^, weight loss of <20%, and ulcerations exceeding 5 mm in length and 1 mm in depth), the experimental endpoint (tumor regression or better survival in the treatment groups), or when the well-being of the mice were impaired. Technical staff were blinded during treatment injections and tumor measurements. All measurements of tumor volume and weight were plotted in GraphPad Prism, and curves were edited in Illustrator (Adobe).

### Ex vivo localization of injected ADCs

0.5 mg/kg of ADC-MMAE and ADC-DXd were injected intravenously in the tail veins of A375 WT or NCI-H1975 tumor-bearing mice with a tumor size of 100–150 mm^3^. Tumor, kidney, spleen, heart, liver, lung, muscle, and plasma were harvested from mice 24 h after injection.

The tissues were weighed and added to Precellys Lysing Kit tubes (Precellys, #432-0142), with lysis buffer, consisting of RIPA buffer (Fisher Scientific, #89900) with protease inhibitor cocktail (Roche, #11836170001), at a ratio of 1:6 w/v tissue: buffer. Mechanical homogenization was performed with the Precellys 24 Tissue Homogenizer (Berton Instruments), using an “Elastic” program (680 rpm, 4 × 20 seconds, 45 s break). Tissues were transferred from the lysing kit tubes to vials, and benzonase (Sigma Aldrich, #E1014) was added to the tissues at a concentration of 35 µU/µl for 30 min at 37 °C. Determinations of protein concentrations were done using a Pierce BCA Protein Kit Assay (Thermo Fisher, #23225).

For Western Blot, 30 µg of tissue homogenate and 0.2 µl plasma in 10 µl RIPA buffer were loaded per lane on a polyacrylamide gel, and detection of the injected ADCs in loaded samples was performed by Western Blot using methods described above.

### Immunohistochemistry detection of injected proteins

A375 tumor-bearing mice were injected intravenously with 0.5 mg/kg of ADC-MMAE. Tumor, liver, and kidney were harvested 24 h after injection and fixed for 24 h at 4% paraformaldehyde before transferring to 70% ethanol and paraffin embedding. Tissue blocks and tissue slides (3 µm thickness) were prepared by the Histolab Core facility at the University of Copenhagen.

Tissue slides were baked for 30 min at 65 °C to melt the paraffin before proceeding with a deparaffinization and rehydration steps consisting of sequential incubation in Xylene Substitute (Tissue-Tek®, Sakura, #94-1466), absolute ethanol (Honeywell, #02860), 96% Ethanol (VWR, #20824.385) 70% Ethanol (VWR, #83801.360), tap water and distilled ultra-pure water. Then, a heat-mediated antigen retrieval was followed, where tissues were boiled for 64 min at 97 °C–100 °C in 10 mM sodium citrate, pH6 buffer, before cooling down to reach RT. Blocking of endogenous peroxidase for 20 min, 37 °C with BLOXALL^®^ Endogenous blocking solution (Vector Laboratories, #SP-6000) was followed by a protein blocking for 1 h, RT using 2.5% Normal Horse Serum (Vector Laboratories, #S-2012-50). Tissues were subsequently washed 3 × 5 min with PBST, and incubated overnight, 4 °C with Rabbit mAb V5 (D3H8Q, Cell Signaling, #13202) diluted 1:400 in Signal Stain antibody diluent (Cell Signaling, #8112). Following 3 × 5 min washes with PBST, ImmPRESS HRP Horse Anti-Rabbit IgG Polymer reagent (Vector Laboratories, #MP-7401) was added and incubated 30 min at 37 °C. Signal development was performed with ImmPACT DAB EqV Substrate (Vector Laboratories, #SK-4103) until the desired stain intensity developed, and the reaction was stopped in tap water. Counterstain with hematoxylin solution (Merck, #MHS32) was performed before tissue dehydration, drying, and mounting using VectaMount Express Mounting medium (Vector Laboratories, #H-5700-60). Imaging was done on a Zeiss Axio Z1 automated slide scanner (×20 magnification, 0.8 NA objective) and analysis on Zen blue software (Zeiss).

### Rat toxicology

Sprague Dawley male rats (6 weeks) were ordered from Janvier. Housing of the animals and experiments was conducted at the Department for Experimental Medicine at the University of Copenhagen. The animals were grouped and housed in individually ventilated cage racks with two animals per cage. The rats were maintained in a 12 h light cycle with a humidity level of 45–65% at 22 °C, handled in accordance with FELASA Rodent Health Surveillance program, and were provided with enrichment opportunities. A preliminary pilot study with weight measurements as the primary endpoint was performed to identify the maximum tolerated dose (MTD) in rats, and the dosage for the repeat dose study was selected to be below the MTD to ensure safety. Two rats were assigned per group and were dosed with either vehicle, 0.5 mg/kg, or 5 mg/kg of Vartumab ADC-MMAE (DAR = 1) successively, once a week for a total of 4 weeks. A recovery period of 2 weeks was set up to observe the recovery of toxicity. The primary endpoint of the study was weight measurements and animal behavior, which were recorded at least twice a week. Secondary endpoints were (i) hematology studies which were done twice— during treatment after two doses and subsequently two weeks post-recovery, (ii) biochemistry studies done with the post-recovery samples, (iii) histopathology. Hematological analysis was performed in accordance with established guidelines using the Element HT5 veterinary hematology analyzer (Heska, USA). Each parameter was measured twice and plotted individually. Serum samples for biochemistry studies were harvested from the full blood and outsourced to Veterinært Diagnostisk Laboratorium (Institute for Klinisk Veterinærmedicin). Kidney and liver panels from the results and parameters with similar units are plotted together. For histopathology, formalin-fixed paraffin-embedded tissue sections were prepared and stained with hematoxylin and eosin (H&E).

### Statistics

For in vitro assays, data are shown as mean ± standard error of the mean (SEM) from technical triplicates. No biological triplicates or inferential statistics were applied. In vivo studies included five animals per treatment group. In studies with less than five treatment groups, individual tumor growth curves are shown. In studies with more treatment groups, tumor volumes are presented as group means ± SEM for visual clarity. Tumor volume data were analyzed using linear mixed-effect models to account for repeated measurements over time. Treatment group, day, and interaction were included as fixed effects, while mouse ID was included as a random effect to account for inter-mouse variability. Models were fitted using the lmer() function from the lmerTest package in R (version 4.5.1), and *p*-values were calculated using Satterthwaite’s approximation.

## Results

### Generation of Vartumab ADCs

Combining the correct antibody and linker-payload for ADCs is paramount since it affects hydrophobicity and, therefore, potentially efficacy and toxicity of the compound. Based on previously published data, Vartumab conjugated to a microtubule-inhibiting payload (MMAE) through a cleavable vc linker demonstrated tumor eradication in several cell-derived xenograft (CDX) and patient-derived xenograft (PDX) models with no evident adverse effect [29]. Topoisomerase inhibitors, such as DXd, have shown strong therapeutic effects in animal models and a relatively large therapeutic window in human clinical studies [32]. Both vc-MMAE and ggfg-DXd are used in market-approved ADCs [3]. Thus, we set out to evaluate how these linker-payloads performed in combination with Vartumab.

**Fig. 1:**
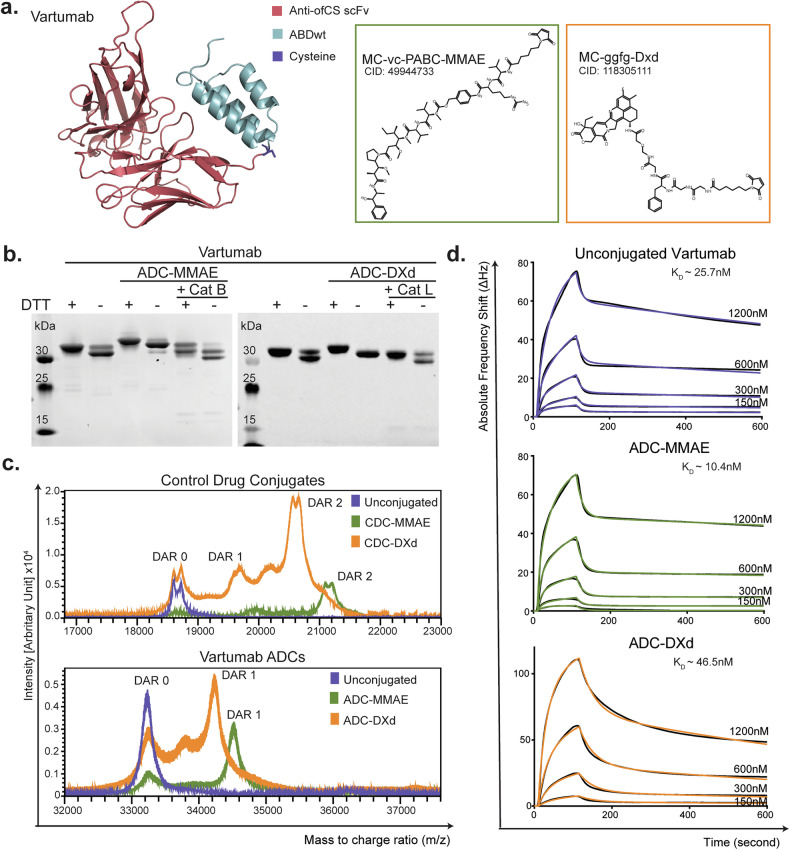
Generation of Vartumab ADCs. **a** Overview of i) Vartumab, ii) maleimidocapryol-valine-citrulline-PABC-MMAE (vc-MMAE) and iii) maleimidocapryol-glycine-glycine-phenylalanine-glycine-DXd (ggfg-DXd). **b** SDS-PAGE analysis of Vartumab conjugated to vc-MMAE or ggfg-DXd through maleimide binding to free cysteines, before and after incubation with recombinant cathepsin B (Cat B) or cathepsin L (Cat L) prior to loading on the gel. All samples were run at 1ug/well ± DTT. **c Upper panel:** Intact mass spectrometry spectra of control proteins in the native state (blue; DAR = 0)) and conjugated to vc-MMAE (green; DAR = 1.8) or ggfg-DxD (orange; DAR = 1.9). **Lower panel:** Vartumab either unconjugated (blue; DAR = 0) or conjugated to vc-MMAE (green; DAR = 0.9) or ggfg-DxD (orange; DAR = 0.8). **d** Sensograms representing binding to ofCS carrying proteoglycan of Vartumab and ADCs for protein concentration varying between 150 and 1200 nM tested on Attana biosensor. Colored curves are experimental points, and binding constant (*K*_D_) was determined using a 1:2 fitting model as depicted by black curves.

Vartumab was designed as a single-chain variable fragment (scFv) genetically fused to an albumin-binding peptide (ABD) for half-life extension purposes. Furthermore, we engineered a single free cysteine at the C-terminal of the scFv to allow for site-directed linker-payload conjugation. Generation of Vartumab ADCs with either maleimido-caproyl-vc-PABC-MMAE (Compound Identification: 49944733) or maleimido-caproyl-ggfg-DXd (Compound Identification: 118305111) were done by conjugating maleimide activated linker-payloads to a free cysteine on Vartumab (ADC) or an irrelevant ABD containing control protein with two free cysteines (CDC) (Fig. 1a). Conjugation was confirmed by SDS-PAGE showing an upward band shift of the ADCs as compared to unconjugated proteins (Fig. 1b and Supplementary Fig. 1a–c) with the shift being most pronounced for the larger vc-MMAE linker-payload compared to the ggfg-DXd linker. The low molecular weight species observed in Fig. 1b is believed to be distinct oxidation species that moves differently under non-reducing conditions, stemming from the cell line used to produce Vartumab. CDCs also showed an upward band shift, confirming conjugation (Supplementary Fig. 1a). The vc linker attached to MMAE is sensitive to cysteine cathepsins, including cathepsin B, K, L, and S [33]. The ggfg linker attached to DXd is also sensitive to cysteine cathepsins, including cathepsin L, but also to hydrolysis [34]. These linker strategies give an additional tumor specificity of an ADC, as the tumor microenvironment (TME), in contrast to peripheral blood, has a high expression of cathepsins and a low pH [35]. Here, we confirmed payload release from the linker-payloads by incubating the ADC with cysteine proteases. SDS-PAGE analysis showed a downward shift in the ADC-MMAE and ADC-DXd sizes after 16 h of incubation with cathepsin B or cathepsin L, respectively, validating linker cleavage and release of the toxin payload (Fig. 1b and Supplementary Fig. 1b, c). The stability of the ADCs was next tested over a 168 h time period using two conditions: (i) in a storage buffer containing L-cysteine, mimicking competition for maleimide conjugation through Retro-Michael reaction, and (ii) in human plasma, where albumin has been shown to induce Retro-Michael reaction [36] (Supplementary Fig. 1d, e). The SDS-PAGE analysis of samples collected from storage buffer at different time points reveals stable ADCs, with no detectable degradation products over one week (Supplementary Fig. 1d). ADCs incubated in human plasma for one week demonstrated high stability, as confirmed by western blot analysis (Supplementary Fig. 1e). These experiments show a favorable stability profile of the ADCs.

Intact mass spectrometry quantified the drug-to-antibody ratio (DAR), by analyzing ADC populations and comparing the protein mass with and without the attached linker-payloads. DARs were calculated by dividing the mass change following conjugation by the linker-payload mass. CDCs did not achieve complete conjugation for any of the payloads, indicated by the presence of a population matching the size of the unconjugated protein (Fig. 1c, *upper panel*). The MMAE conjugation primarily produced CDCs with a DAR of 2, along with smaller populations of DAR1 and DAR0. In contrast, the DXd conjugation had a broader distribution, with some DAR2 populations, and more DAR1 and DAR0 populations. As these populations were not seen on the SDS-Page (Fig. 1b), it is likely due to the sample preparation procedure, where a low pH of the TFA buffer could hydrolyze the ether bond in the ggfg linker. Based on this analysis, the DAR was determined to be 1.9 for the CDC-MMAE and 1.8 for the CDC-DXd. A similar pattern was observed for the Vartumab ADCs in which a sub-population of unconjugated protein remained (Fig. 1c*, lower panel*). The DAR0 peak was higher in the DXd conjugate compared to the MMAE conjugate, believed to be due to the low pH sample preparation buffer. For these ADCs, the DARs were determined to be 0.9 for the ADC-MMAE, and 0.8 for the ADC-DXd.

Next, we tested whether the toxin conjugation affected the binding to ofCS. We compared the binding kinetics of unconjugated protein, ADC-MMAE, and ADC-DXd to immobilized ofCS using an Attana quartz microscale biosensor that measures affinity interactions through weight changes. The conjugation minimally affected binding to ofCS, with a slight increase of ADC-MMAE affinity towards the ligand (from ~25.7 nM to ~10.4 nM), and a slight decrease for ADC-DXd (from ~25.7 nM to ~46.5 nM) (Fig. 1d).

These data show that we were able to produce Vartumab ADCs and CDCs with both linker-payloads at close to full conjugation to the available cysteines. All drug conjugates were sensitive to cleavage by relevant proteases, but still stable in buffer and plasma. Furthermore, the resulting Vartumab ADCs maintained binding kinetics to ofCS as compared to unconjugated protein.

### Specificity of Vartumab ADCs to human cancer cells and tissues

**Fig. 2:**
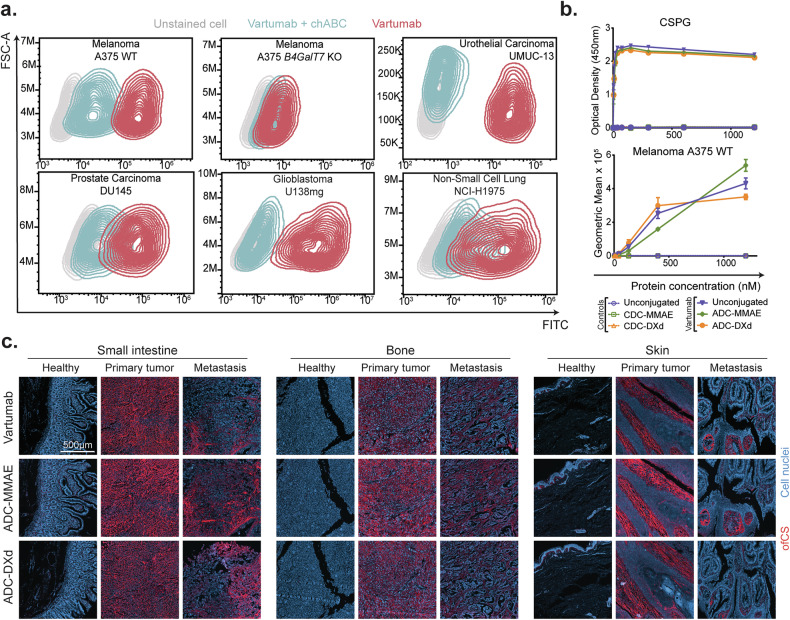
Specificity of Vartumab ADCs to human cancer cells and tissues. **a** Binding of Vartumab (red) to a panel of epithelial human cancer cell lines at 300 nM (melanoma A375 wild-type (WT) or *B4GALT7* knock out (KO)), or 1200 nM for (prostate adenocarcinoma DU145 and PC-3, glioblastoma U138mg, non-small cell lung adenocarcinoma NCI-H1975 and bladder urothelial carcinoma UMUC-13), compared to specificity control (chondroitinase ABC treated cells (blue) and secondary antibody control (light gray). **b Upper panel:** Binding (OD450) of unconjugated protein and ADCs, binding to recombinant CSPG [0–1200] nM detected through V5 or His tag in ELISA. **Lower panel:** Melanoma A375 binding of unconjugated protein and ADCs [0–1200] nM detected through FITC-labeled anti-V5 or His tag antibody. Binding was measured as relative geometric mean fluorescence intensity (MFI) and normalized against secondary antibody controls. All samples were run in triplicate (*n* = 3). **c** Immunofluorescence scans of fixed and paraffin-embedded needle biopsies of healthy tissue, primary tumor, and metastasis from the small intestine, bone, and skin, stained with DAPI (blue) and 25 nM Vartumab, ADC-MMAE, or ADC-DXd detected with anti-V5-Alexa Fluor 647 antibody (red).

We next expanded the in vitro testing to validate that functionalizing the antibody fragment into an ADC did not affect ofCS-specificity. Previously, we have tested an extensive panel of cancer cell lines for Vartumab binding, confirming binding across various cell lines [29]. This panel was expanded here with human cancer cell lines, including prostate carcinoma (DU145), urothelial carcinoma (UMUC-13), glioblastoma (U138-mg), and non-small cell lung carcinoma (NCI-H1975). We confirmed binding of Vartumab with high CS specificity, as the signal disappears after chondroitinase ABC (chABC) enzymatic treatment (Fig. 2a). Additionally, the protein control did not show any binding to these cell lines (Supplementary Fig. 2a). All tested cell lines are listed in Supplementary Table 1, and the gating strategy is detailed in Supplementary Fig. 2b.

Using ELISA, we first demonstrated that the ADCs retained binding to chondroitin sulfate proteoglycans (CSPG) at levels comparable to the unconjugated antibody, while the CDCs had no binding (Fig. 2b*, upper panel*). Similarly, the albumin binding peptide retained full binding to human serum albumin for both ADCs and CDCs (Supplementary Fig. 2c*, left panel*). Next, we tested that the ADCs maintained ofCS specificity to cancer cells using flow cytometry and confirmed binding to the human melanoma cancer cell A375 WT, comparable to the unconjugated protein (Fig. 2b*, lower panel*). Notably, the ADCs retained ofCS specificity as demonstrated with low binding to ofCS knockout cell line A375 *B4GALT7* KO in which glycosaminoglycan production was abolished by knocking out the *B4GALT7* gene coding for one of the key enzymes synthesizing the tetra-linker (Supplementary Fig. 2c*, right panel*).

To examine the specificity of the ADCs for human cancer tissue, we compared the binding of ADCs and Vartumab to thin needle biopsies using immunofluorescence on a multi-organ human tissue microarray (TMA) (Fig. 2c and Supplementary Figs. 3 and 4). Pixel quantification for positive staining to each biopsy confirmed that unconjugated Vartumab had minimal signal in healthy and benign tissues, while most tissue specimens exhibited ofCS binding in the primary tumors (Supplementary Fig. 5a). Vartumab differentiated healthy, malignant, and metastatic tissues in small intestine, bone, and skin. This specificity was maintained following conjugation with the different linker-payloads (Fig. 2c). Following conjugation, both ADCs showed a slight but limited increase in binding to healthy biopsies (Fig. 2c and Supplementary Figs. 4 and 5a). This increase was not surprising since the hydrophobicity introduced by the ADCs’ payload could increase off-target binding [37].

Collectively, these data show that binding is retained after conjugation with linker-payloads and that Vartumab ADCs can be used to target primary tumors as well as disseminated metastases within a broad range of malignancies.

### Localization and tissue penetration of Vartumab ADCs in vivo

**Fig. 3:**
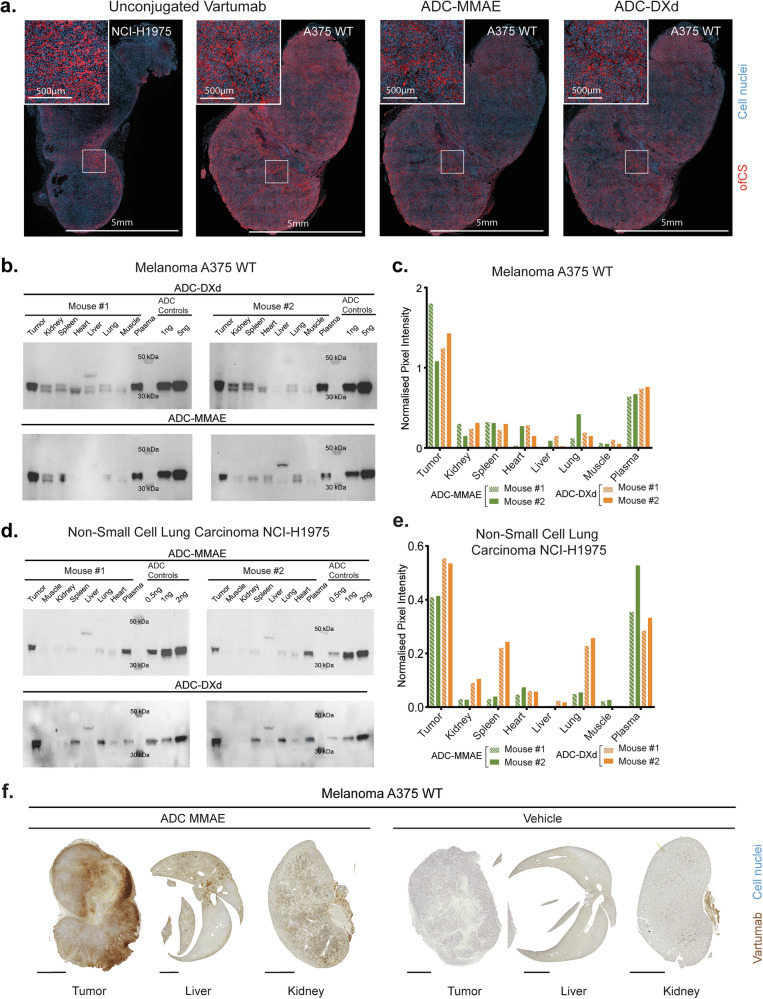
Localization and tissue penetration of Vartumab ADCs in vivo. **a** Immunofluorescence scans of fixed and paraffin-embedded cell-derived-xenografted tumors of melanoma A375 WT or non-small cell lung carcinoma NCI-H1975 models, stained with DAPI (blue) and 25 nM Vartumab, ADC-MMAE, or ADC-DXd, detected with anti-V5-Alexa Fluor 647 antibody (red). **b** Western blot of injected Vartumab ADCs in melanoma A375 tumor-bearing mice. 0.5 mg/kg ADC-MMAE or ADC-DXd was injected in mice, and tissues were collected after 24 h. The tissues collected were tumor, kidney, spleen, heart, liver, lung, muscle, and plasma. 30 µg of homogenized tissue were run along with non-injected ADC controls at 1 and 2 ng. ADCs were detected through V5-tag. **c** Quantification of signals from western blots of (**b**). analyzed in Image Lab and normalized to the non-injected control lanes. **d** Western blot of injected Vartumab ADCs in non-small cell lung NCI-H1975 tumor-bearing mice. 0.5 mg/kg ADC-MMAE or ADC-DXd was injected in mice, and tissues were collected after 24 h. The tissues collected were tumor, kidney, spleen, heart, liver, lung, muscle, and plasma. 30 µg of homogenized tissue were run along with non-injected ADC controls at 0.5, 1, and 2 ng. ADCs were detected through V5-tag. **e** Quantification of signals from western blots of (**d**). analyzed in Image Lab and normalized to the non-injected control lanes. **f** Immunohistochemistry detection of V5-tagged Vartumab in tumor, liver, and spleen of A375 model. Mice were injected with the ADC-MMAE or PBS vehicle, and organs were collected 24 h after and detected through their V5 tag. Displayed liver and kidney correspond to Vartumab-injected specimens. The scale bar indicates 2000 µm.

To assess the binding of Vartumab ADCs in tumor tissue, mice were engrafted with melanoma A375 or non-small cell lung carcinoma NCI-H1975 cells. When tumors reached a volume of 200 mm^3^, animals were injected with 0.5 mg/kg of ADC and euthanized after 24 h; tissues were fixed and embedded in paraffin. Immunofluorescence staining of the tissues confirmed Vartumab binding to the NCI-H1975 and A375 tumors with a tendency for reduced binding following conjugation, irrespective of the linker-payload (Fig. 3a). Binding was abolished by enzymatic chABC treatment, validating again the CS specificity of Vartumab and derived ADCs (Supplementary Fig. 6a). In NCI-H1975 tumors, staining was less intense but showed a similar binding pattern to the stromal compartment of the tumor (Fig. 3a).

We have previously shown that Vartumab upon intravenous (i.v.) injection localizes to tumors of tumor-bearing mice with limited accumulation in other tissues [29]. However, since toxin conjugation can affect pharmacodynamics, we compared tissue distribution of unconjugated Vartumab and the two Vartumab ADCs. Mice with A375 melanoma tumors or non-small cell lung carcinoma NCI-H1975 tumors were i.v. injected with 10 µg of V5 epitope-tagged Vartumab ADCs and euthanized after 24 h. Tumor tissue, plasma, and various organs, including kidney, spleen, heart, liver, and lung, were collected for ex vivo analysis. Samples were analyzed on Western Blot, detecting the injected proteins by the V5-tag and assessing tissue biodistribution through pixel quantification. Ponceau staining of the membrane following protein transfer validated that equal amounts of tissue homogenates were loaded in each lane, ensuring that signal intensities could be reliably compared between samples (Supplementary Fig. 6b, c). Analyses indicated that the ADCs remained in circulation 24 h after injection, as testified by the signals detected in plasma (Fig. 3b, e and Supplementary Fig. 7a, b). Both ADC-MMAE and ADC-DXd effectively localized to the A375 melanoma tumors, with uptake levels 5-7x higher than in the other organs (Fig. 3b, c and Supplementary Fig. 7a). In the NCI-H1975 lung model, the ADC-MMAE tumor uptake was comparable to the melanoma model, but the ADC-DXd had some distribution to the spleen and lungs, potentially attributed to unspecific accumulation as immunofluorescence staining showed a slight increase in accumulation to healthy tissue for the ADC-DXd (Fig. 3d, e and Supplementary Fig. 5a and 7b). Excluding the lungs and spleen, the ADC-DXd tumor uptake was 6 times higher than in the other organs. Next, we investigated the penetration of the ADC-MMAE into a melanoma A375 tumor by detecting the IV injected ADC by immunohistochemistry 24 h after injection. ADC-MMAE showed complete tumor penetration beyond tumor vessels compared to a vehicle-injected tumor, with limited localization and no penetration in healthy organs (Fig. 3f). This demonstrates that ADCs retained the ofCS tumor specificity in animals, specifically localized and fully penetrated into the tumor with minimal uptake into other organs.

### In vitro efficacy of Vartumab ADCs

**Fig. 4:**
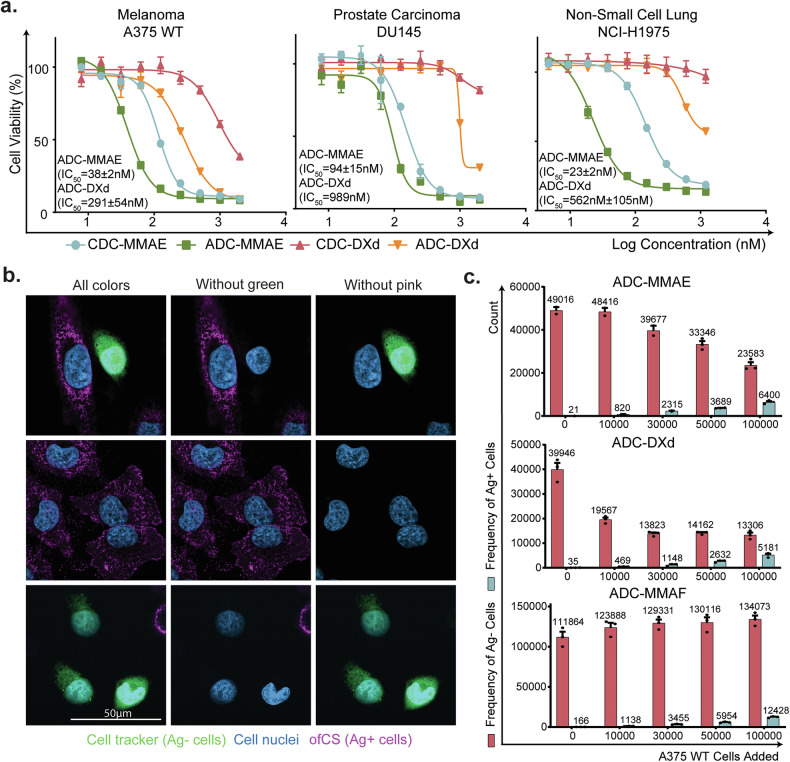
In vitro efficacy of Vartumab ADCs and their mode of action. **a** Cytotoxicity assay on melanoma A375 WT, prostate DU145, and non-small cell lung NCI-H1975 cancer cell lines incubated with ADC at [0–1200] nM concentrations. Each ADC was run in triplicate (*n* = 3), error bars indicate variance in cell viability between triplicates **b** Immunofluorescence staining of melanoma A375 WT cells (Ag+) and A375 *B4GALT7* KO cells (Ag−) with DAPI (blue), and 25 nM Vartumab, detected with Anti-V5 Alexa Fluor 647 (pink). A375 *B4GALT7* KO cells were labeled with cell tracker (green) before co-culturing. **c** Cell counts of A375 *B4GALT7* KO cells co-cultured with A375WT cells and incubated with ADCs for 72 h. Ag− cells were stained with cell tracker green and seeded at 100,000 cells/well. Ag+ cells were added at increasing concentrations (from 0 to 100,000). Cells were treated with Vartumab ADC > IC_80_ (MMAE = 100 nM, DXd = 600 nM, and MMAF = 50 nM). All ADC treated wells were run in triplicate. ADC MMAF was used as a control for a non-permeable payload.

We next assessed in vitro cytotoxicity of the ADCs on a panel of human cancer cell lines (A375 melanoma, NCI-H1975 lung, and DU145 prostate). For all cell lines, Vartumab ADCs exhibited greater cytotoxicity than the controls (CDCs) (Fig. 4a). The MMAE-conjugate was consistently more potent than the DXd-conjugate with IC_50_ values of 38 nM, 94 nM, and 23 nM for ADC-MMAE compared to 291 nM, 989 nM, and 562 nM for ADC-DXd in A375, NCI-H1975, and DU145 cell lines, respectively. Furthermore, the ADC-MMAE had similar potency in A375 and NCI-H1975 cell lines, while ADC-DXd had the highest potency in A375. Vartumab is designed to be a monovalent fragment to facilitate tumor penetration into ofCS dense tumors. The high amounts of ofCS in tumors compensate for the lower avidity by retaining the scFv in the tumor, as also demonstrated in Fig. 3f. Thus, it was anticipated that in vitro IC_50_ killing for Vartumab ADC is higher than a bivalent IgG ADC [31].

To validate that both MMAE and DXd toxins could induce cell death through bystander effects [14], we set up a co-culture experiment using melanoma A375 WT ofCS positive (Ag+) cells alongside an ofCS knockout (Ag−) line, A375 *B4GALT7* KO, as validated in flow (Fig. 2a and Supplementary Fig. 2c*, right panel*). In an immunofluorescence staining experiment, we mixed the *B4GALT7* KO (Ag−) cells labeled with a green fluorescent dye with WT (Ag+) cells and first confirmed that Vartumab only bound to the Ag+ cells (Fig. 4b). We then seeded Ag- cells with an increasing concentration of Ag+ cells and examined how treatment with Vartumab ADCs affected cell populations by counting live cells in flow cytometry (Supplementary Fig. 8a). All seeding conditions were run in triplicates along with non-treated controls to confirm that one cell line does not outgrow the other (Supplementary Fig. 8b). For this experiment, a Vartumab ADC-MMAF was produced to serve as a negative control, since the polar nature of the payload prevents it from penetrating the cell membrane’s lipid bilayer, thereby not allowing this ADC to act through a bystander effect. Quantification showed that in the presence of MMAE and DXd ADCs, killing of Ag- cells is enhanced in a concentration-dependent manner with the addition of Ag+ cells (Fig. 4c), whilst ADC-MMAF served as a negative control and demonstrated effective killing of Ag+ cells but no dose-dependent killing of Ag- cells.

### In vivo Vartumab ADC efficacy in mice and toxicity in rats

**Fig. 5:**
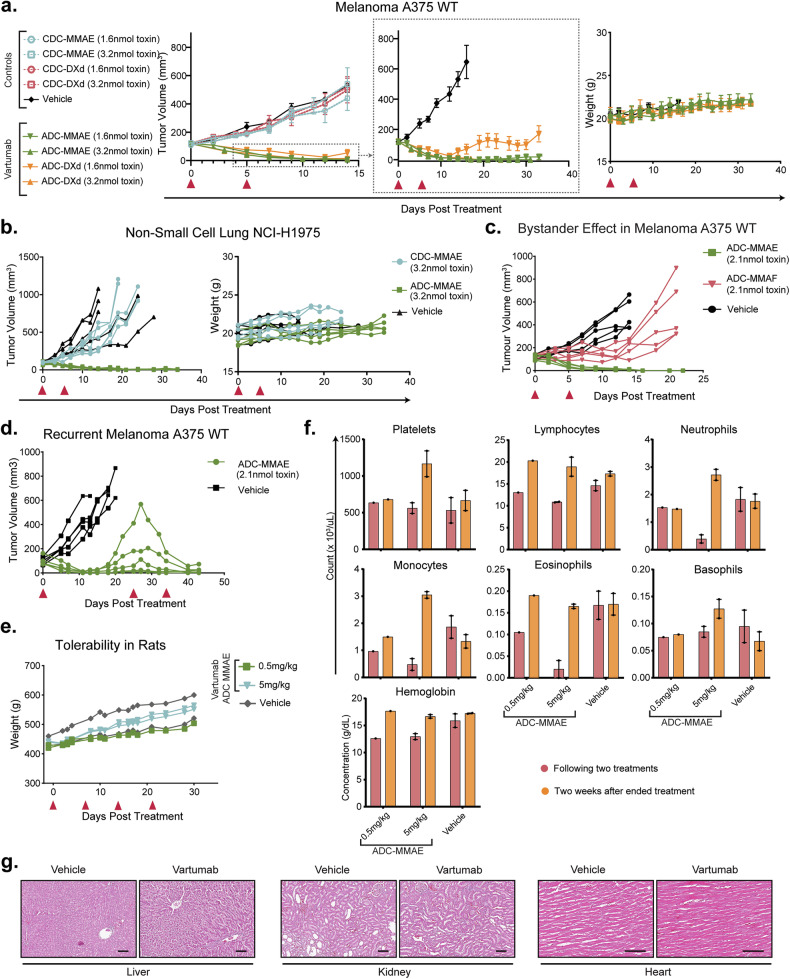
Vartumab ADC efficacy and safety in vivo. **a** Tumor burden (mean ± SEM mm^3^) of all groups until first death (*n* = 5 each group) where melanoma A375-bearing mice were treated with CDCs and ADCs. Vehicle is PBS. Red arrows indicate days of treatment. Vartumab groups are also shown in focus, for dose differentiation in tumor burden. Weights are shown for Vartumab groups and the vehicle. At the end of the study, ADC MMAE groups had 4/5 and 5/5 tumor-free, while ADC DXd groups had 0/5 and 5/5 tumor-free, for 1.6 nmol toxin and 3.2 nmol toxin, respectively. All ADC-treated groups had significant tumor reduction compared to their corresponding controls (*p* < 0.0001 for all group x day interaction terms). Comparison between 1.6 nmol toxin ADC-MMAE and ADC-DXd revealed greater efficacy of ADC-MMAE (*p* < 0.0001), while at 3.2 nmol toxin there was no significant difference between ADC-MMAE and ADC-DXd (*p* = 0.681). **b** Tumor burden (mm^3^) and weights of non-small cell lung NCI-H1975-bearing mice treated with CDC-MMAE and ADC-MMAE (*n* = 5 each group). Vehicle is PBS. Red arrows indicate days of treatment. At the end of the study, the ADC-MMAE group had 1/5 tumor-free. ADC-MMAE showed significantly improved efficacy over CDC-MMAE (*p* < 0.0001). **c** Tumor burden of melanoma A375-bearing mice treated with ADC-MMAE and ADC-MMAF at 2.1 nmol toxin (*n* = 5 each group). Vehicle is PBS. Red arrows indicate days of treatment. At the end of the study ADC-MMAE group had 5/5 tumor-free, and the ADC-MMAF group had 0/5 tumor-free. ADC-MMAF was significantly less effective than ADC-MMAE (*p* < 0.0001). **d** Tumor burden (mm^3^) of melanoma A375-bearing mice treated with ADC-MMAE at 2.1nmol toxin (*n* = 5 each group). Vehicle is PBS. Red arrows indicate days of treatment. At the end of the study, the ADC-MMAE group had 1/5 tumor-free. **e** Tolerability of Vartumab ADC-MMAE assessed by measuring the weight of rats treated at 0.5 mg/kg or 5 mg/kg for multiple doses (*n* = 2 per group, one rat in 0.5 mg/kg was euthanized due to improper vein injection). Vehicle is PBS. **f** Hematology profile from rats treated with 0.5 mg/kg or 5 mg/kg Vartumab ADC-MMAE from (**e**). Following two treatments and 2 weeks after the end of treatment. **g** Representative hematoxylin and eosin staining of liver, kidney, and heart of rats following treatment with 5 mg/kg Vartumab ADC MMAE or vehicle. The scale bar represents 100 µm. For **a–c** statistical analyses were performed using a linear mixed-effects model in *R*, modeling tumor volume over time as a function of treatment group, time, and their interaction, with mouse ID as a random effect. Significance was assessed using Satterthwaite’s method (lmerTest).

ADC-DXd has a higher IC_50_ value compared to ADC-MMAE (Fig. 4a), which is also reflected in previous studies reporting lower systemic toxicity of DXd, but in turn requiring higher ADC dosing to reach similar anti-tumor efficacy [4]. To examine the in vivo potency of the ADCs, female BALB/c nu/nu mice were xenografted subcutaneously with melanoma A375 cells and treated twice with our drug-conjugates five days apart. Dosages were set at equimolar amounts of toxin from previous literature [31] and set at 1.6 nmol and 3.2 nmol toxin per treatment, thereby adjusting for differences in DAR. Both Vartumab ADCs were able to regress the tumor, while the CDCs had no effect and grew similarly to tumors in mice receiving the vehicle (Fig. 5a). The tumors regressed completely in response to ADC-MMAE and ADC-DXd at a concentration of treatment of 3.2 nmol toxin, while at a concentration of 1.6 nmol toxin, ADC-DXd led to a partial response and a complete response for ADC-MMAE. Body weights were not affected by any of the treatments, suggesting a lack of evident adverse effects (Fig. 5a). Based on ofCS staining (Fig. 3a) and in vitro potency (Fig. 4a), it was decided to test Vartumab ADC-MMAE in a second cell-derived xenograft model of non-small cell lung cancer (NCI-H1975) in BALB/c nu/nu mice, dosing at 3.2nmol toxin. While the control-drug conjugate and vehicle groups did not affect tumor growth, ADC MMAE was able to completely regress the tumors, without causing any weight loss (Fig. 5b). To examine the level of in vivo bystander effect of Vartumab we tested head-to-head ADC-MMAE and ADC-MMAF as used in Fig. 4c. In vitro cytotoxicity assay of the two ADCs showed that they are equally potent at 45 nM (Supplementary Fig. 9a). While both ADCs were well tolerated in terms of weight changes (Supplementary Fig. 9b), there were clear differences in efficacy as only ADC-MMAE with the membrane permeable payload was able to completely regress the tumor. ADC-MMAF was able to stall tumor growth during initial treatment, indicating that while some cells may have been killed by internalization, the less permeable payload was not able to kill supporting cells upon release of toxin (Fig. 5c). Down-regulation of the target following exposure to treatment is one mechanism that can cause ADCs to fail to elicit a good response. Therefore, we have tested this by xenografting female BALB/c nu/nu mice subcutaneously with melanoma A375 cells and treated once with the ADC-MMAE at 2.1 nmol toxin. Following an initial therapeutic response, tumor regrowth occurred, at which point another treatment cycle was commenced with 2.1 nmol toxin ADC-MMAE (Fig. 5d). Tumors regressed with two treatments, indicating that ofCS was not down-regulated after the initial treatment.

To form a baseline for GLP toxicological and dose finding studies, we evaluated the initial safety of the Vartumab ADC-MMAE in a repeat dose toxicology study in rats at two doses—0.5 mg/kg and 5 mg/kg. One rat in the 0.5 mg/kg group was euthanized due to a drug unrelated tail injury post second injection. The initial safety profile was assessed through weight monitoring and hematology profiles. All rats gained weight, indicating that there were no obvious or acute adverse effects after the two dosages (Fig. 5e). Blood profiles from samples collected after the first two ADC treatments and two weeks post-recovery revealed increased hemoglobin, and platelet counts following ended treatment, suggesting that at high doses the treatment could have caused repression which is being compensated for, but further studies are needed to determine this (Fig. 5f). Counts of lymphocytes, neutrophils, monocytes and eosinophils decreased during treatment for the higher dose, but their numbers returned to baseline after recovery and were comparable to vehicle group (Fig. 5f). Further analysis of biochemistry markers, including urea nitrogen, kidney profiling and liver profile indicators, showed similar levels to the vehicle group within normal ranges (Supplementary Fig. 9c). However, the levels of creatine kinase (CK) were lower for 0.5 mg/kg of ADC-MMAE as compared to the vehicle. Histopathology examination of liver, kidney, and heart tissues of the rats showed no indication of ADC-induced pathology in these organs (Fig. 5g). Since no signs of muscle atrophy were observed from the HE tissue staining, reasons for variability in CK levels were ascribed to rat handling and sampling, also reported in the literature [38]. Together, these data show that repeat dosing of Vartumab ADC conjugated with vc-MMAE is well tolerated in rats at a minimum 5 mg/kg dose level.

## Discussion

ofCS was first discovered in the placenta through its interaction with the malaria parasite protein VAR2CSA, utilized by the parasite to mediate placental adhesion and immune evasion. Later, it was found that ofCS reappears in malignant tissue across tumor types and is absent or minimally expressed in normal organ tissues other than fetal tissue [20]. Although therapeutic formulations of VAR2CSA demonstrated good efficacy in preclinical tumor models [20], it proved challenging to transition a malaria protein for clinical development. Following phage display antibody panning on ofCS, an antibody fragment (Vartumab) was identified that retained a high tumor specificity with limited binding to normal tissues. Our accumulated data points to ofCS not only being present on the surface of the malignant cells but also secreted and presented as a modification to proteoglycans, such as collagens and versican, present in the malignant extracellular matrix, and importantly also present on tumor stromal cells like cancer-associated fibroblasts (CAF) [20, 29]. This broad, yet selective, tumor-associated binding makes ofCS a potential ideal target for an ADC.

Traditionally, ADC efficacy has been understood to rely primarily on antigen internalization into target cells followed by payload release. However, this mechanism requires antigen expression on both cancer and stromal cells to achieve a comprehensive therapeutic effect. In contrast, targeting an ECM-associated antigen, such as ofCS presents as a compelling alternative which enables payload release directly into the tumor microenvironment, leveraging the bystander effect to eliminate both malignant and supportive cells, independent of internalization. To achieve this kind of efficacy, the design should incorporate a cleavable linker and a payload with a strong bystander effect, aligning with a conceptual shift toward ECM-targeted ADCs, which exploit the tumor microenvironment to broaden anti-tumor activity beyond internalization-dependent mechanisms.

In the present study, we evaluated Vartumab conjugated with two clinically validated linker-payloads (vc-MMAE and ggfg-DXd). To extend half-life of the Vartumab scFv, we genetically fused the ADC with an albumin-binding peptide and further engineered the ADC to have a single free cysteine for site-specific conjugation through maleimide chemistry. Both ADCs were fully conjugated to the engineered cysteine and demonstrated toxin release upon enzymatic cleavage. Importantly, both ADCs maintained the specificity of Vartumab following toxin conjugation, including retained binding to recombinant CSPG, albumin, cancer cells, and thin human needle biopsies across multiple tumor types, with low binding to healthy tissues or benign tumors. The increased hydrophobicity of the molecule resulted in a small increase in the binding to healthy tissue compared to unconjugated Vartumab [39]. To validate the design of the Vartumab ADC for giving a bystander effect, co-culture experiments with ofCS knockout (Ag−) and ofCS WT (Ag+) cells were performed. This data demonstrated that both linker-payloads showed Ag+ concentration-dependent killing of Ag- cells, indicating bystander exposure. These in vitro findings were consolidated in an in vivo xenograft model comparing ADCs with: vc-MMAE that, after release, can readily diffuse across membranes and thus give a bystander effect; and vc-MMAF, which, upon cleavage, results in a less permeable payload unable of producing bystander effect [40]. Both ADCs were equally potent in vitro, whereas the ADC-MMAE resulted in complete in vivo tumor regression, and the ADC-MMAF only stalled tumor growth, supporting that, to have complete tumor regression, an ofCS targeting ADC needs to be designed with a linker-payload able to generate bystander effect. Clinical studies demonstrate that the bystander effect enhances ADC efficacy even when the target is confined to cancer cells and absent from the extracellular matrix or stroma, as exemplified by HER2-targeting therapies [41]. For the Vartumab ADC, the observed distribution of ofCS suggests that toxin release may occur at least partly in the stromal and extracellular space, leading to cell killing with the bystander effect. This could lead to some leakage into the circulation, as well as potential toxicity to adjacent normal cells, but it may also enhance therapeutic efficacy by effectively eliminating antigen-negative tumor cells, as well as eliminating microtumor satellite cells within the surrounding margin. Importantly, no unexpected systemic toxicity has been observed in our preclinical models, suggesting that limited free MMAE is circulating.

The anti-tumor effects of MMAE and DXd have been thoroughly investigated, with MMAE being more potent than DXd in vitro and in vivo, albeit some cell lines show resistance to MMAE [31, 42–45]. The data shows that both the vc-MMAE and ggfg-DXd linker-payloads can be used for making anti-ofCS ADCs with bystander effects while maintaining the specificity to ofCS. However, from clinical studies, it is evident that the therapeutic window is larger for DXd compared to MMAE payloads, and the trend is thus to dose higher with DXd ADCs but also to increase the DARs, while keeping it low for MMAE conjugates [46, 47]. In line with this, we found that ADC-MMAE exhibited potent cell killing against melanoma A375, non-small cell lung carcinoma NCI-H1975, and prostate carcinoma DU145, while ADC-DXd was 10 to 20-fold less potent in vitro. Translating this into in vivo models, both ADCs provided a powerful anti-tumor effect in the melanoma A375 model, but the dose differentiation illustrated the potency differences between the payloads. Here, ADC-MMAE had high anti-tumor efficacy at low dose, while ADC-DXd only showed complete regression at the higher dose. This potency difference has also been reported by others, in which DXd conjugates are effective at higher doses compared to MMAE conjugates [31]. Furthermore, the ADC-MMAE exhibited a strong anti-tumor response in a non-small cell lung NCI-H1975 model, despite the fact that immunofluorescence staining showed less ofCS expression in this model as compared to the melanoma model, highlighting the therapeutic potential of a stromal targeting ADC with bystander effect. Importantly, it was also shown that ofCS is not down-regulated following treatment with the ADC and that the tumors could be treated again, indicating that ofCS may be essential for promoting tumor growth.

The results presented here were obtained with the scFv fused to an albumin-binding domain for plasma half-life extension. This non-human peptide could be immunogenic upon repeat dosing in humans and interfere with albumin binding. Before production for clinical efficacy trials, the scFv should be half-life extended through Fc fusion or PEGylation. To support decision on target populations for the ADC phase I, II trials, Vartumab is currently being tested in an immuno-PET/CT study in 16 cancer patients designed as a basket study in solid tumors, including both rare and common malignancies (Trial ID: NCT06645808). This study aims to support the pan-cancer potential of Vartumab, but could also indicate if some cancer types show better Vartumab tumor uptake.

In conclusion, we produced two Vartumab ADCs with vc-MMAE and ggfg-DXd, which retained specific binding to ofCS, in different malignant and metastatic tissues, with limited healthy tissue binding. The linker-payload design of the ADCs was validated by showing bystander killing of Ag- cells, and by tumor efficacy in two CDX models, one with high and one with low ofCS expression. Importantly, we showcased that Vartumab requires a linker-payload which consists of a cleavable linker and a membrane-permeable payload to achieve potent anti-tumor response through bystander effect, which less permeable payloads are unable to elicit. Finally, ADC-MMAE was also shown to be well-tolerated at a clinically relevant dose with reversible toxicities associated with the MMAE toxin. These findings underscore the potential for a paradigm shift in ADC design—one which challenges the sole reliance on receptor-mediated internalization and instead embraces dual targeting of cancer and stromal cells to disrupt the tumor network more comprehensively.

## Supplementary information

Supplementary Material

Generation of Vartumab ADCs

Vartumab specificity in vitro

Vartumab binding to multi-organ human tissue microarray

Vartumab ADCs bind to multi-organ human tissue microarray

Quantification of Vartumab and ADCs’ binding to multi-organ human tissue microarray

Ponceau staining of tissue localization

Localization of Vartumab ADCs in ofCS-expressing tumor models

Bystander killing in vitro

Bystander killing in vivo and toxicology of Vartumab ADC treated rats

Overview of cancer cell lines

Data Set 1

Data Set 2

Data Set 3

## Data Availability

All data relating to the findings in the article are contained in the manuscript and supporting information.
