# Supplementary material for: Preclinical profiling of antibody drug conjugates targeting oncofetal chondroitin sulfate
Source: Cell Death Dis. 2026 Jan 24;17(1):162. doi: 10.1038/s41419-026-08420-x (PMC12877138; doi:10.1038/s41419-026-08420-x)
Supplement: Supplementary file 1 — Supplementary Material [file 41419_2026_8420_MOESM1_ESM.docx]

**Preclinical profiling of antibody drug conjugates targeting oncofetal chondroitin sulfate**

**Authors:** Ann Skafte^1,2^, Elena Ethel Vidal-Calvo^1^, Swati Choudhary^1,2^, Joana Mujollari^1^, Robert Dagil^1,2^, Anne Martin-Salazar^1^, Htoo Zarni Oo^3^, Lara Duvnjak^1,2^, Thor G. Theander^1, 2^, Mads Daugaard^2, 3^, Tobias Gustavsson^1,2^, Ali Salanti^1,2^

**Affiliations:**

^1^Centre for Translational Medicine and Parasitology, Department of Immunology and Microbiology, Faculty of Health and Medical Sciences, University of Copenhagen, Copenhagen University Hospital, Copenhagen, Denmark

^2^VAR2 Pharmaceuticals ApS, Frederiksberg, Denmark

^3^Vancouver Prostate Centre, Vancouver Coastal Health Research Institutes, Vancouver, BC, Canada

Corresponding Authors: Email: [salanti@sund.ku.dk](mailto:salanti@sund.ku.dk)

**Supplementary 1: Generation of Vartumab ADCs**

**a.** SDS-PAGE analysis of control and Vartumab in their unconjugated form or conjugated to a valine-citrulline-MMAE (vc-MMAE), or to glycine-glycine-phenylalanine-glycine-DXd payloads (ggfg-DXd). All samples were run at 1µg of protein per well +/- DTT. **b.** Same as in **a.** with stated proteins were treated with recombinant cathepsin B (Cat B). **c.** Same as in **a.** with stated proteins were treated with recombinant cathepsin L (Cat L). **d.** Buffer stability of ADCs in PBS + 5mM L-Cysteine at time points 0h, 48h and 168h. Unconjugated Vartumab was run at 1ug +DTT to visualize any loss of linker-payload. Time point samples were also run at 1ug +DTT. **e.** Plasma stability of ADCs at time points 0h, 24h, 48h, 120h, 144h and 168h. Plasma samples were loaded at 0.2uL, +DTT and detected by V5-HRP.

**Supplementary 2: Vartumab specificity *in vitro***

**a.** Binding of control protein (red) to a panel of epithelial human cancer cell lines at 300nM (melanoma A375 wild-type (WT) or *B4GALT7* knock out (KO)), or 1200nM for (prostate adenocarcinoma DU145 and PC-3, glioblastoma U138mg, non-small cell lung adenocarcinoma NCI-H1975 and bladder urothelial carcinoma UMUC-13), compared to specificity control (chondroitinase ABC treated cells (blue) and secondary antibody control (light grey). **b.** Gating strategy for cancer cell binding. **c.** **Left panel:** Fluorescence (OD450) of unconjugated protein and ADCs [0-100] nM, binding to recombinant human serum albumin detected through V5 or His tag in ELISA. **Right panel:** Melanoma A375 *B4GALT7* KO binding of unconjugated protein and ADCs [0-1200] nM detected through FITC-labeled V5 or His tag. Binding was measured as relative geometric mean fluorescence intensity (MFI) and normalized against secondary antibody controls. All samples were run in triplicate (*n*=3).

**Supplementary 3: Vartumab binding to multi-organ human tissue microarray**

**a.** Map of commercialized paraffin-embedded tissue-micro-array, BCN1021 (Biomax), representing healthy tissues in purple, benign tissues in green, malignant tissues in pink and metastasis sections in orange from the indicated organs. **b.** Immunofluorescence scan of BCN1021 TMA stained with DAPI (blue) and 25nM unconjugated Vartumab, detected with anti-V5 Alexa Fluor 647 antibody (red). Yellow boxes are depicted in **Fig. 2c**.

**Supplementary 4: Vartumab ADCs binding to multi-organ human tissue microarray**

**a.** Immunofluorescence scan of BCN1021 tissue-micro-array (TMA) stained with DAPI (blue) and 25nM Vartumab ADC-MMAE, detected with anti-V5 Alexa Fluor 647 antibody (red). Yellow boxes are depicted in **Fig. 2c**. **b.** Same as in a. but TMA was stained with Vartumab ADC DXd. Yellow boxes are depicted in **Fig. 2c**.

**Supplementary 5: Quantification of Vartumab and ADCs’ binding to multi-organ human tissue microarray**

**a.** Quantification of red pixels from immunofluorescence staining of paraffin-embedded human multi-organ tissue micro-arrays (BCN1021) stained with 25nM Vartumab, ADC-MMAE or ADC-DXd and detected with anti-V5-Alexa Fluor 647 antibody. Blue dots represent tissue biopsies of healthy organs, while red dots represent malignant sections for the indicated organs.

**Supplementary 6: Ponceau staining of tissue localization**

**a.** Immunofluorescence scans of enzymatic chondroitinase ABC (chABC) treated paraffin-embedded cell-derived xenograft tumors of melanoma A375 WT or non-small-cell-lung NCI-H1975 models. chABC treated tissues were stained with DAPI (blue) 25nM Vartumab, ADC-MMAE or ADC-DXd as indicated and detected with anti V5-Alexa Fluor 647 antibody (red). **b.** Ponceau staining of membrane after transfer of tissues run on SDS-PAGE for western blot in **Fig. 3b**. **c.** Ponceau staining of membrane after transfer of tissues run on SDS-PAGE for western blot in **Fig. 3d**.

**Supplementary 7: Localization of Vartumab ADCs in ofCS expressing tumor models**

**a.** Western blot of injected Vartumab ADCs in melanoma A375 tumor-bearing mice. 10µg ADC-MMAE or ADC-DXd were injected in mice and tissues were collected after 24h. The tissues collected were tumor, kidney, spleen, heart, liver, lung, muscle, and plasma. 30µg of homogenized tissue were run along with non-injected ADC controls at 1 and 2ng. ADCs were detected by V5-tag. Each blot is one mouse. **b.** Western blot of injected Vartumab ADCs in non-small cell lung NCI-H1975 tumor-bearing mice. 10µg ADC-MMAE or ADC-DXd were injected in mice, and tissues were collected after 24h. The tissues collected were tumor, kidney, spleen, heart, liver, lung, muscle, and plasma. 30µg of homogenized tissue were run along with non-injected ADC controls at 0.5, 1, and 2ng. ADCs were detected through V5-tag.

**Supplementary 8: Bystander killing in vitro**

**a.** Gating strategy for bystander study showing first gating on cell populations, single cells, DAPI +/- and Alexa Fluor 488 +/-. **b.** Cell counts of alive A375 WT and *B4GALT7* KO cells from control wells of co-culture experiments. Ag- cells were stained with cell tracker (green) and seeded at 100,000 cells/well. Ag+ cells were unstained and added at increasing concentrations. Cells were incubated with media for the duration of ADC treatment for other wells and counted as the geometric mean fluorescent intensity in DAPI negative populations.

**Supplementary 9: Bystander Killing In Vivo and Toxicology of Vartumab ADC Treated Rats**

**a.** Cytotoxicity assay on melanoma A375 WT cancer cell lines incubated with ADC at [0-1200] nM concentrations. Each ADC was run in triplicate (*n*=3), error bars indicate variance in cell viability between triplicates. **b.** Weight (mean +/- SEM mm^3^) of melanoma A375-bearing mice treated with ADC-MMAE and ADC-MMAF at 2.1nmol toxin (*n*=5 each group). Vehicle is PBS. Red arrows indicate days of treatment. At the end of study, the ADC-MMAE group had 5/5 tumor-free and the ADC-MMAF group had 0/5 tumor-free. **c.** Liver and kidney biochemical profile of rats treated with 0.5mg/kg or 5mg/kg Vartumab ADC-MMAE from **Fig. 5d.** following 2 treatments and 2 weeks after the end of treatment.
