# Supplementary figures and images for: Preclinical profiling of antibody drug conjugates targeting oncofetal chondroitin sulfate
Source: Cell Death Dis. 2026 Jan 24;17(1):162. doi: 10.1038/s41419-026-08420-x (PMC12877138; doi:10.1038/s41419-026-08420-x)

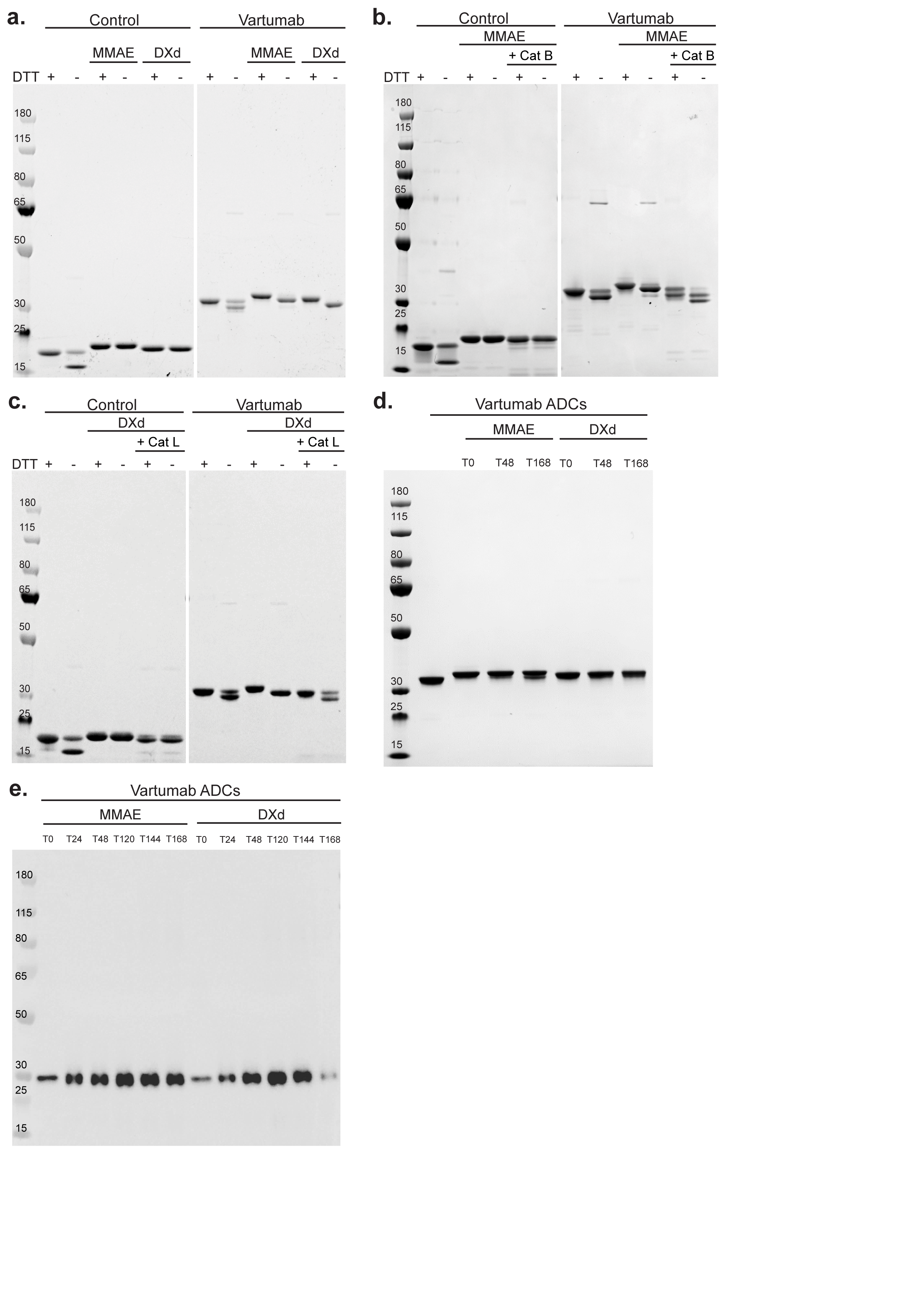

Supplement: Supplementary file 2 — Generation of Vartumab ADCs [file 41419_2026_8420_MOESM2_ESM.tif]

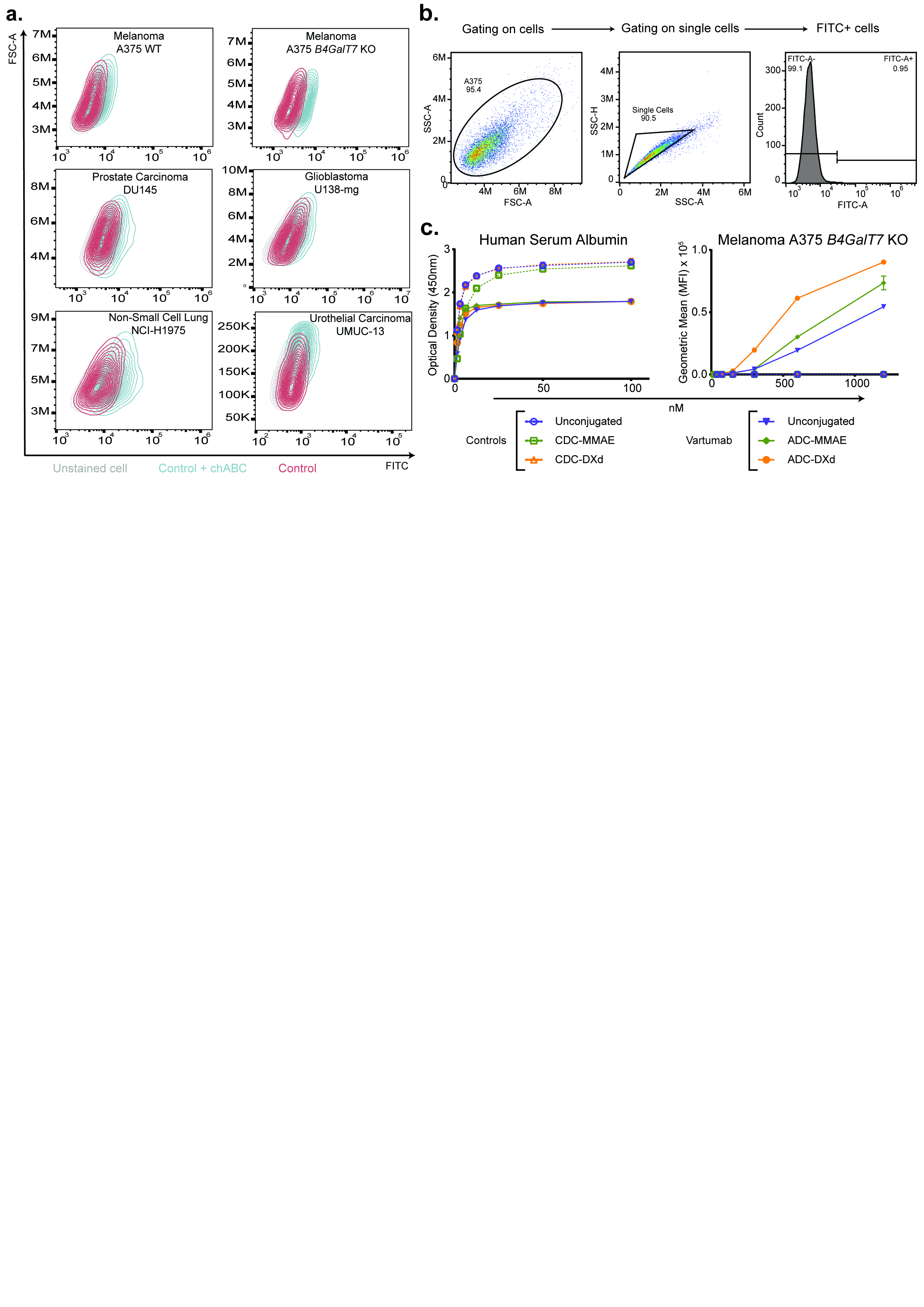

Supplement: Supplementary file 3 — Vartumab specificity in vitro [file 41419_2026_8420_MOESM3_ESM.tif]

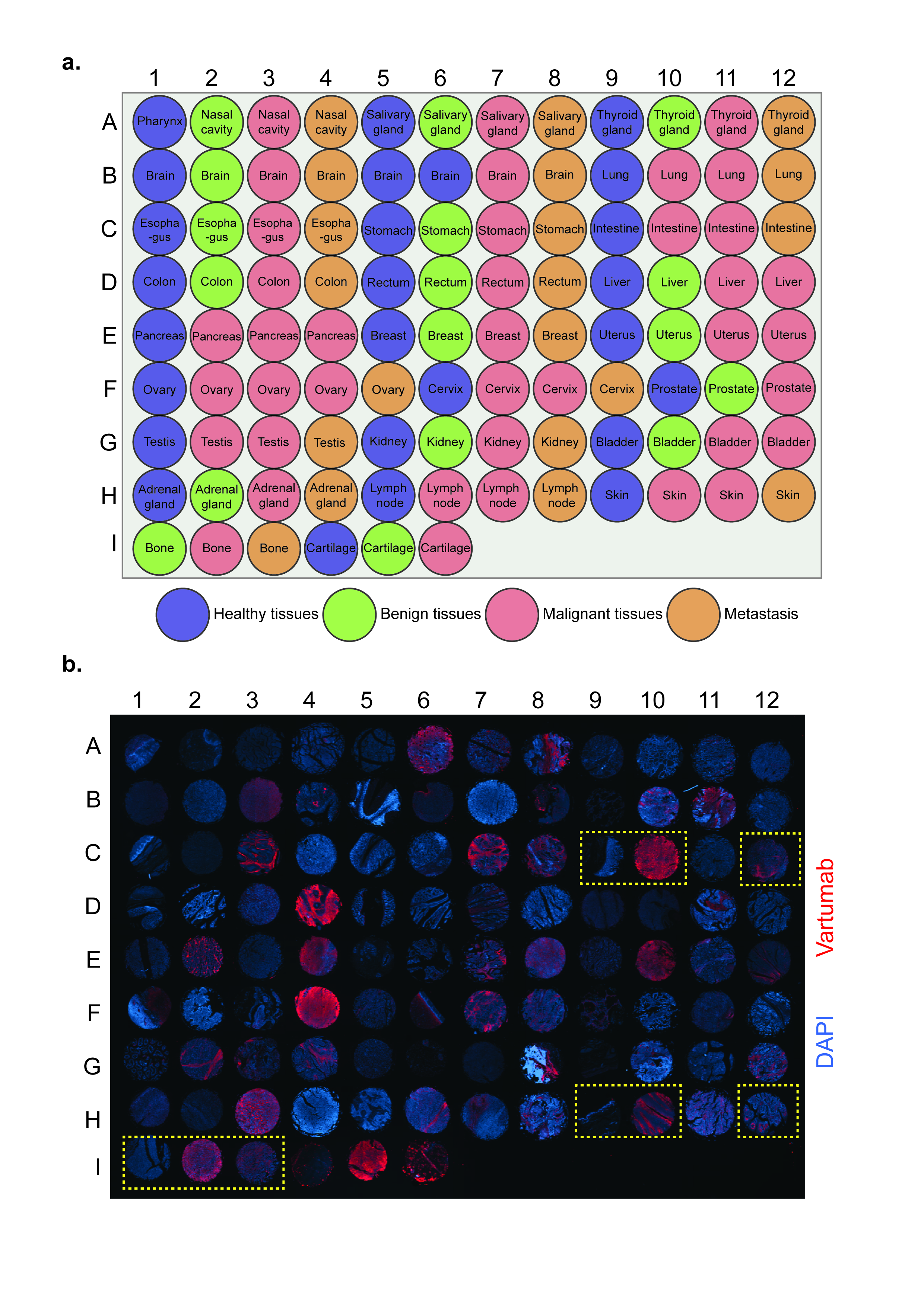

Supplement: Supplementary file 4 — Vartumab binding to multi-organ human tissue microarray [file 41419_2026_8420_MOESM4_ESM.tif]

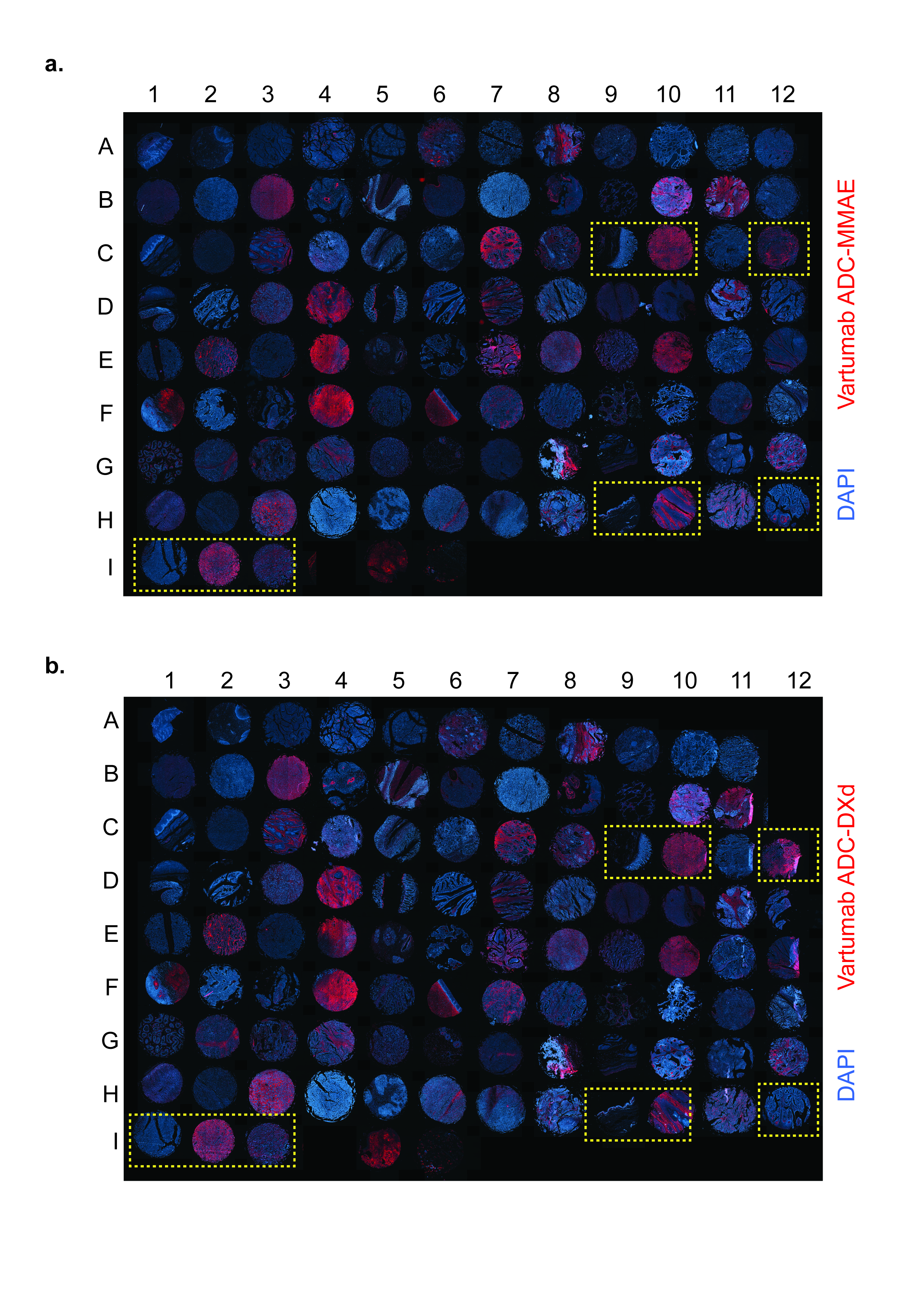

Supplement: Supplementary file 5 — Vartumab ADCs bind to multi-organ human tissue microarray [file 41419_2026_8420_MOESM5_ESM.tif]

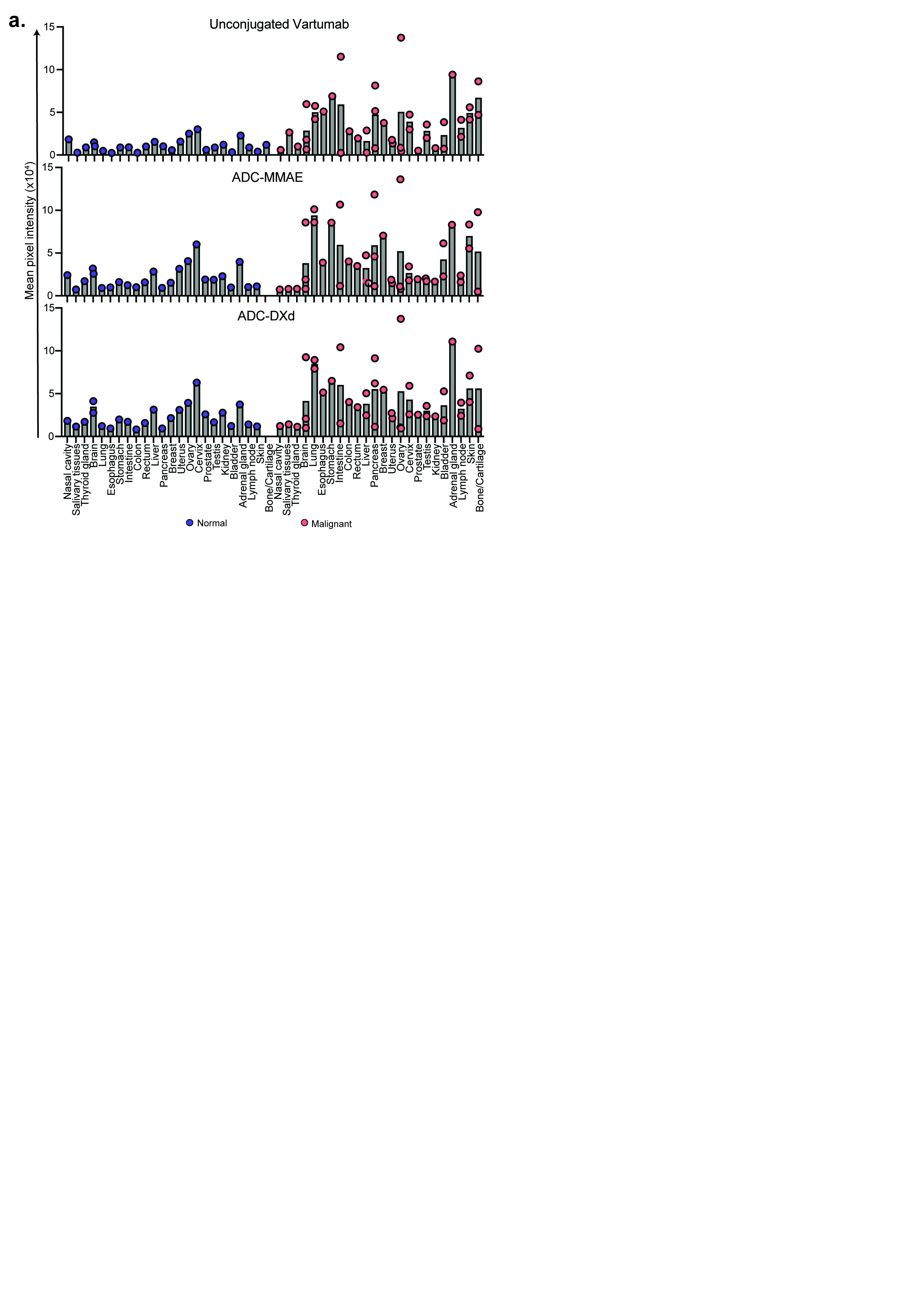

Supplement: Supplementary file 6 — Quantification of Vartumab and ADCs’ binding to multi-organ human tissue microarray [file 41419_2026_8420_MOESM6_ESM.tif]

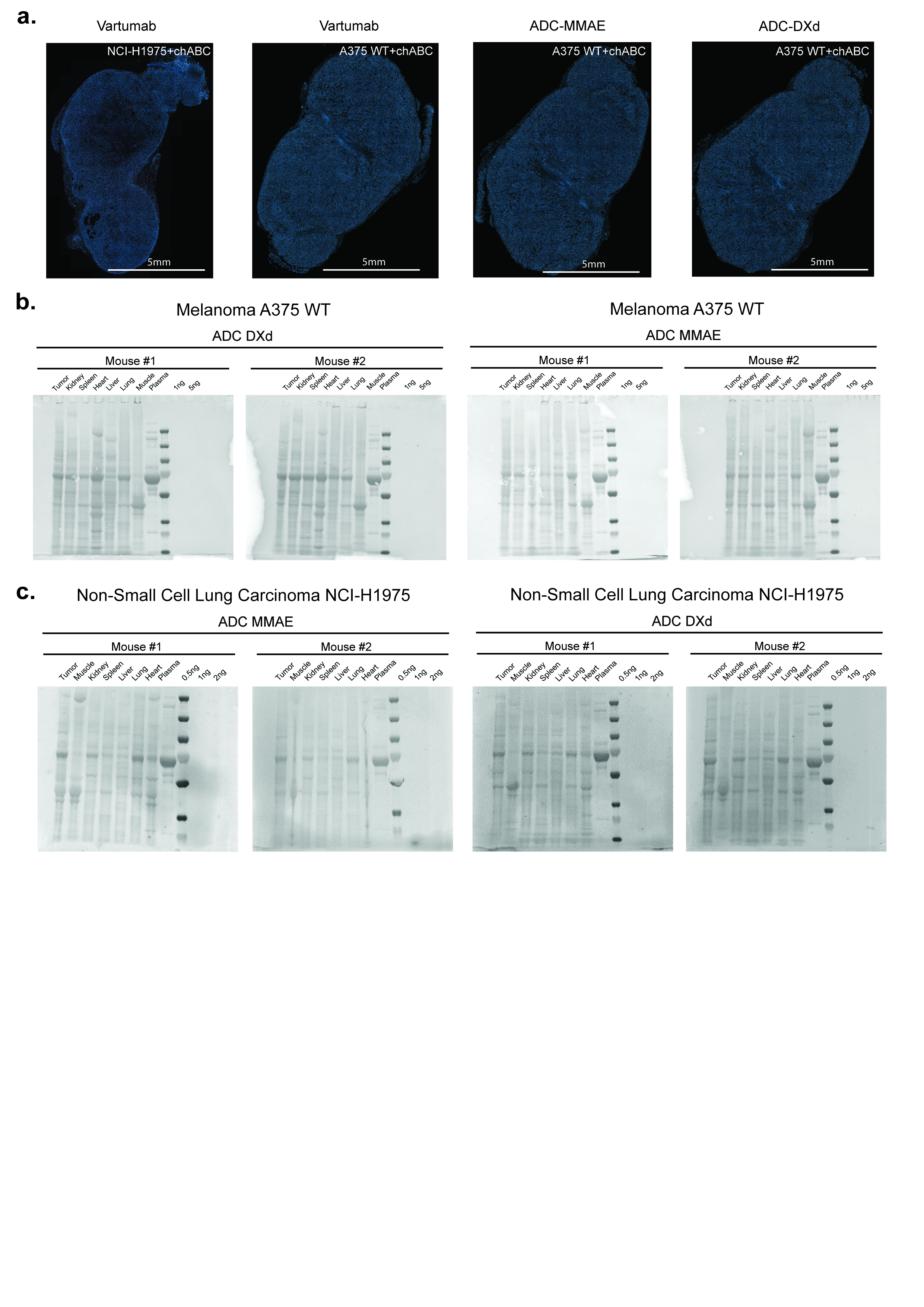

Supplement: Supplementary file 7 — Ponceau staining of tissue localization [file 41419_2026_8420_MOESM7_ESM.tif]

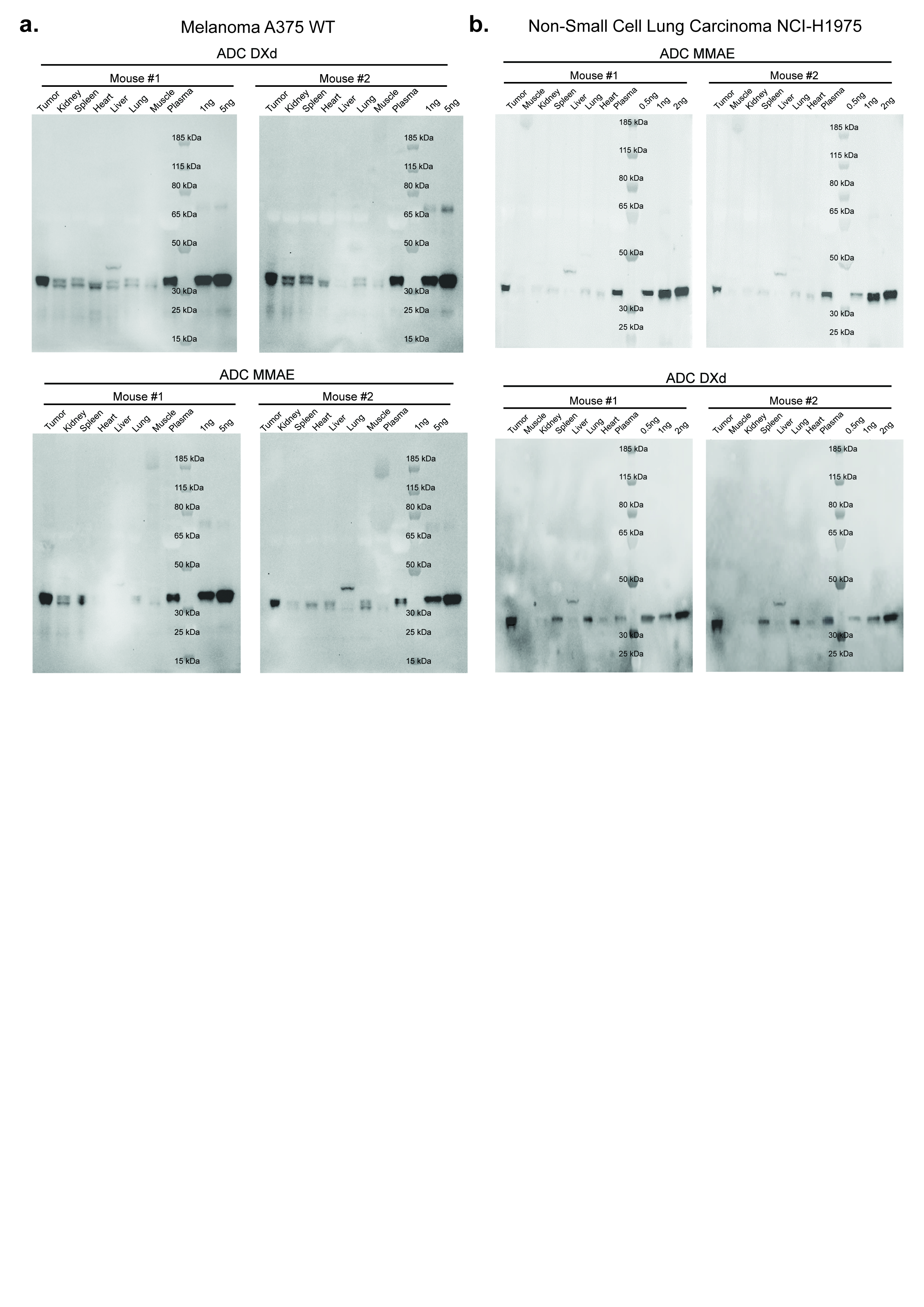

Supplement: Supplementary file 8 — Localization of Vartumab ADCs in ofCS-expressing tumor models [file 41419_2026_8420_MOESM8_ESM.tif]

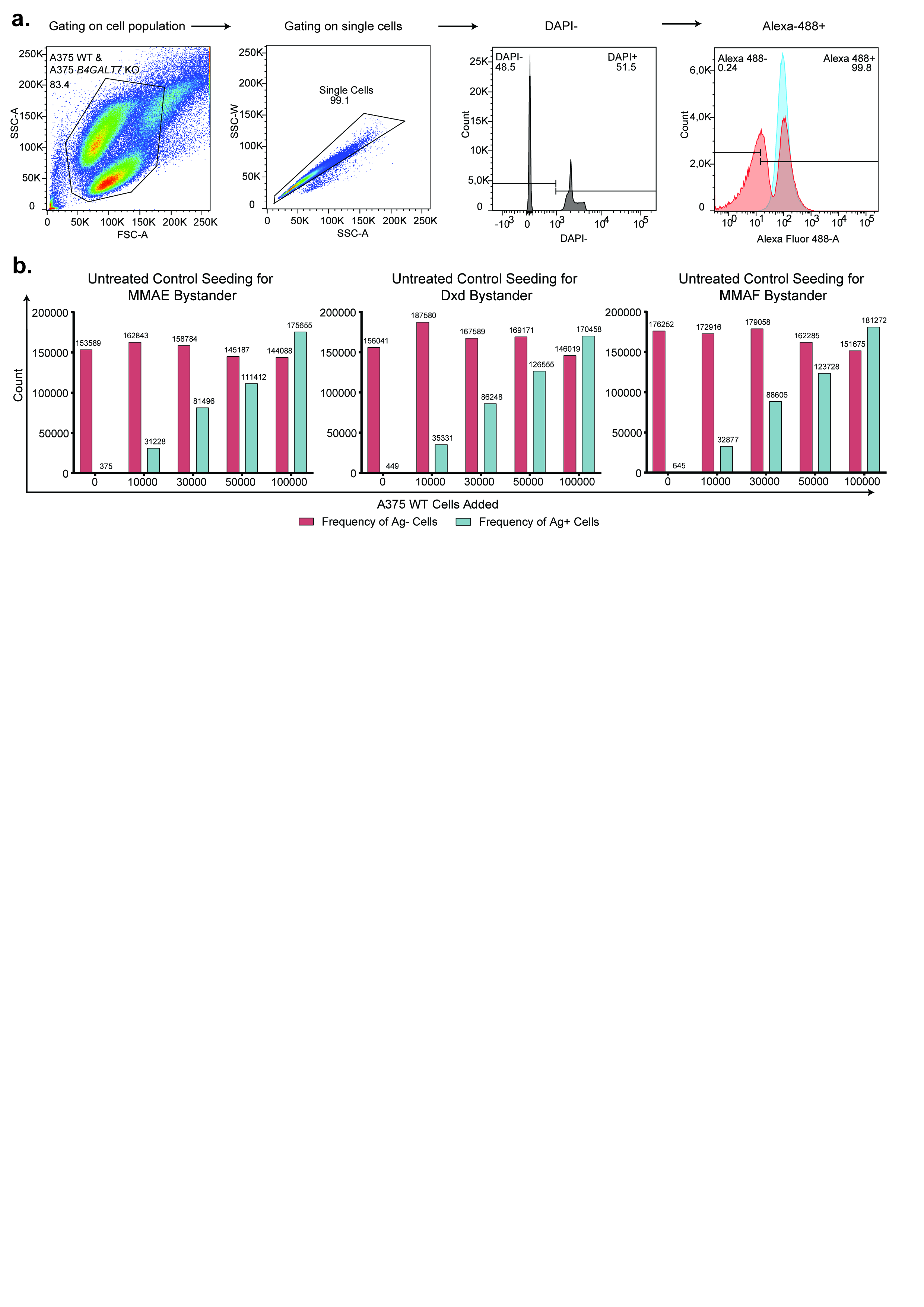

Supplement: Supplementary file 9 — Bystander killing in vitro [file 41419_2026_8420_MOESM9_ESM.tif]

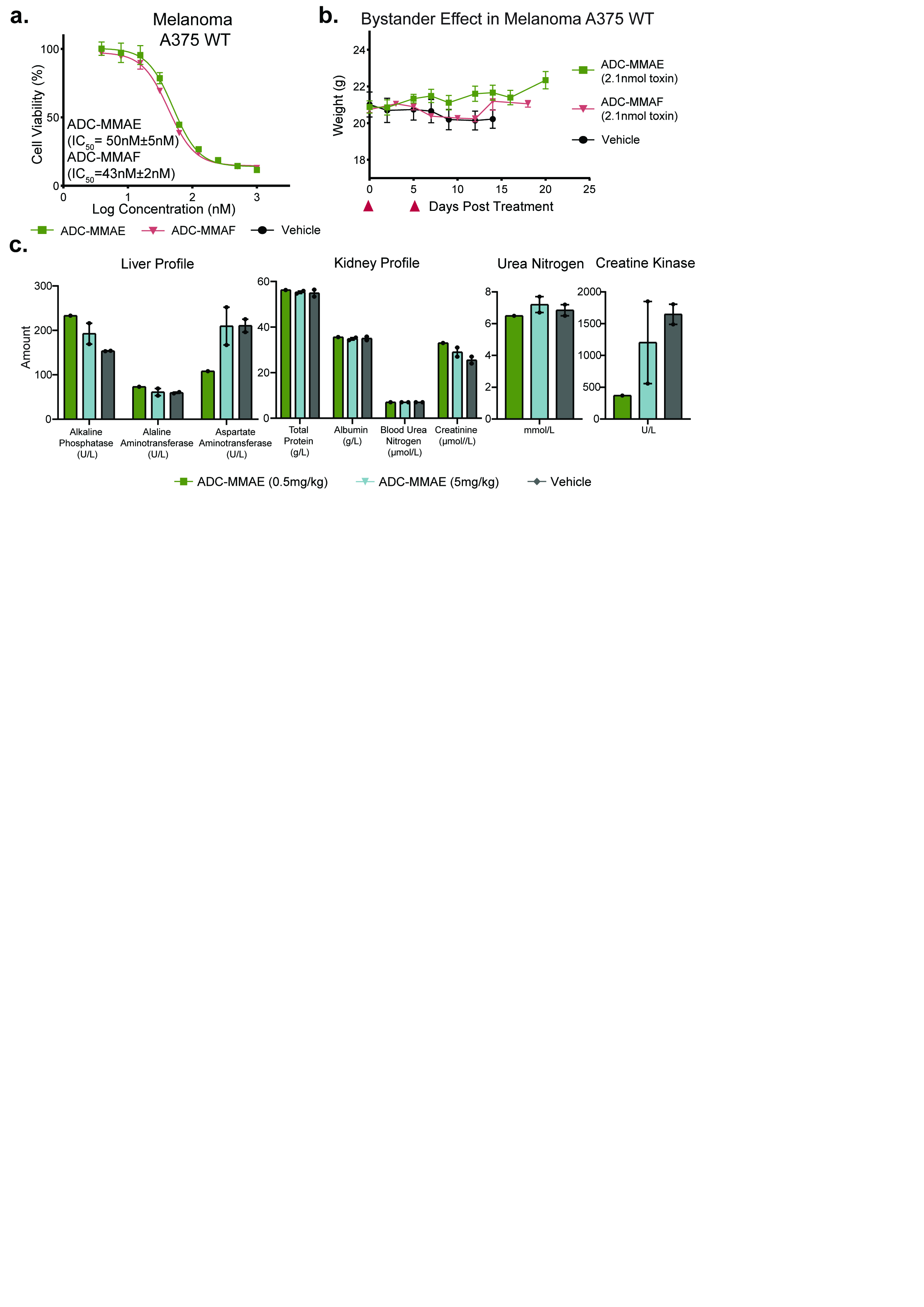

Supplement: Supplementary file 10 — Bystander killing in vivo and toxicology of Vartumab ADC treated rats [file 41419_2026_8420_MOESM10_ESM.tif]

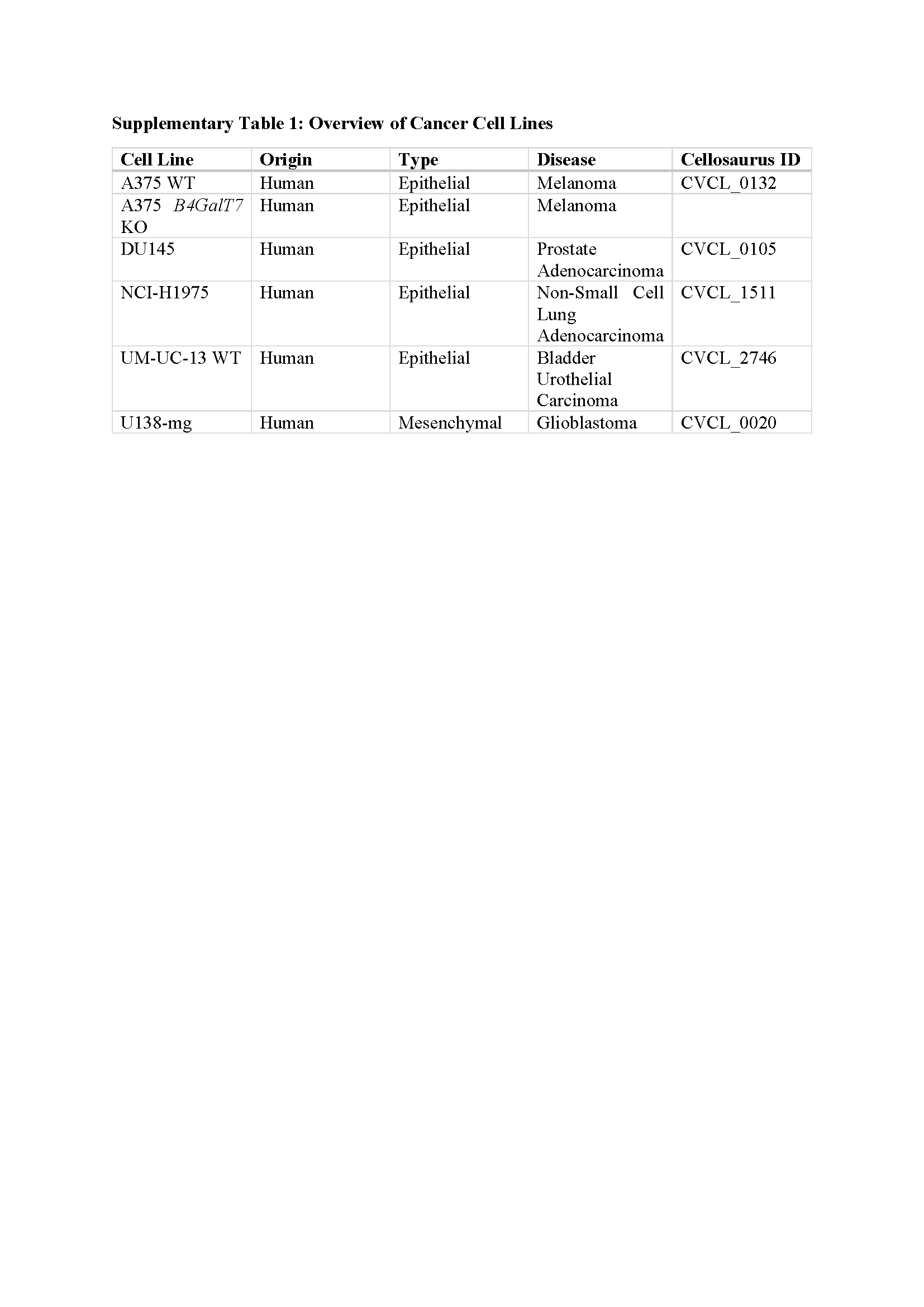

Supplement: Supplementary file 11 — Overview of cancer cell lines [file 41419_2026_8420_MOESM11_ESM.tif]

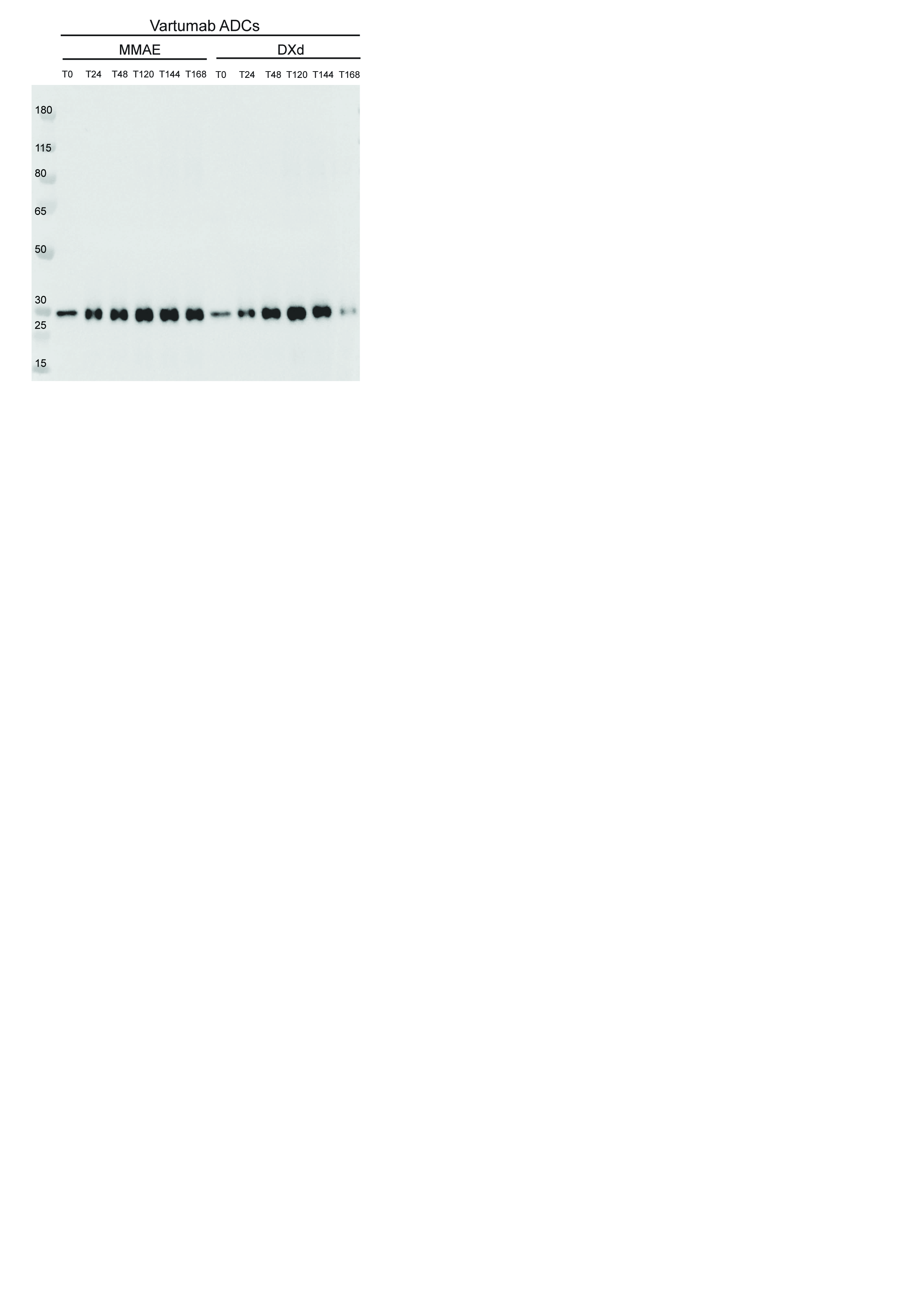

Supplement: Supplementary file 12 — Data Set 1 [file 41419_2026_8420_MOESM12_ESM.tif]

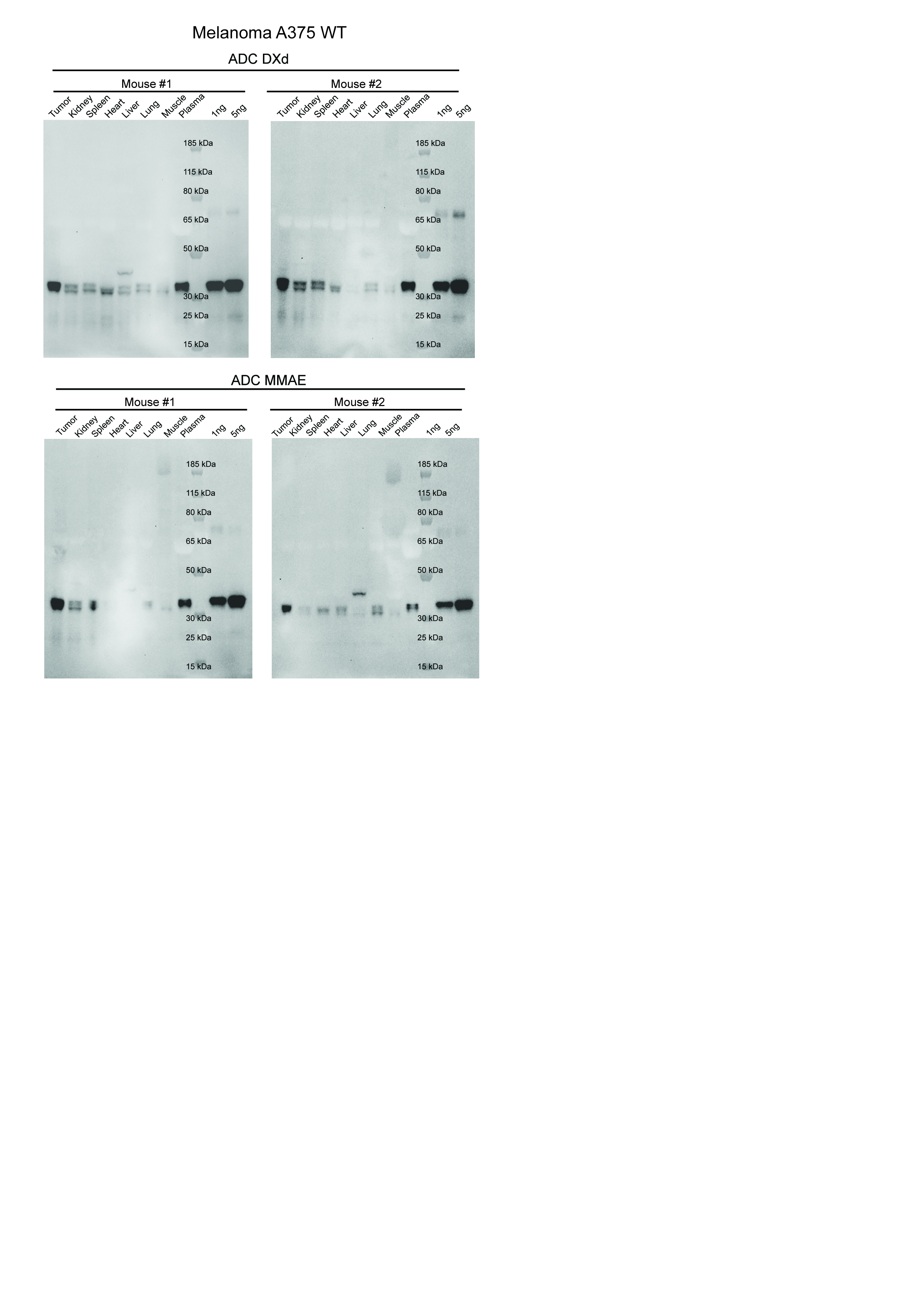

Supplement: Supplementary file 13 — Data Set 2 [file 41419_2026_8420_MOESM13_ESM.tif]

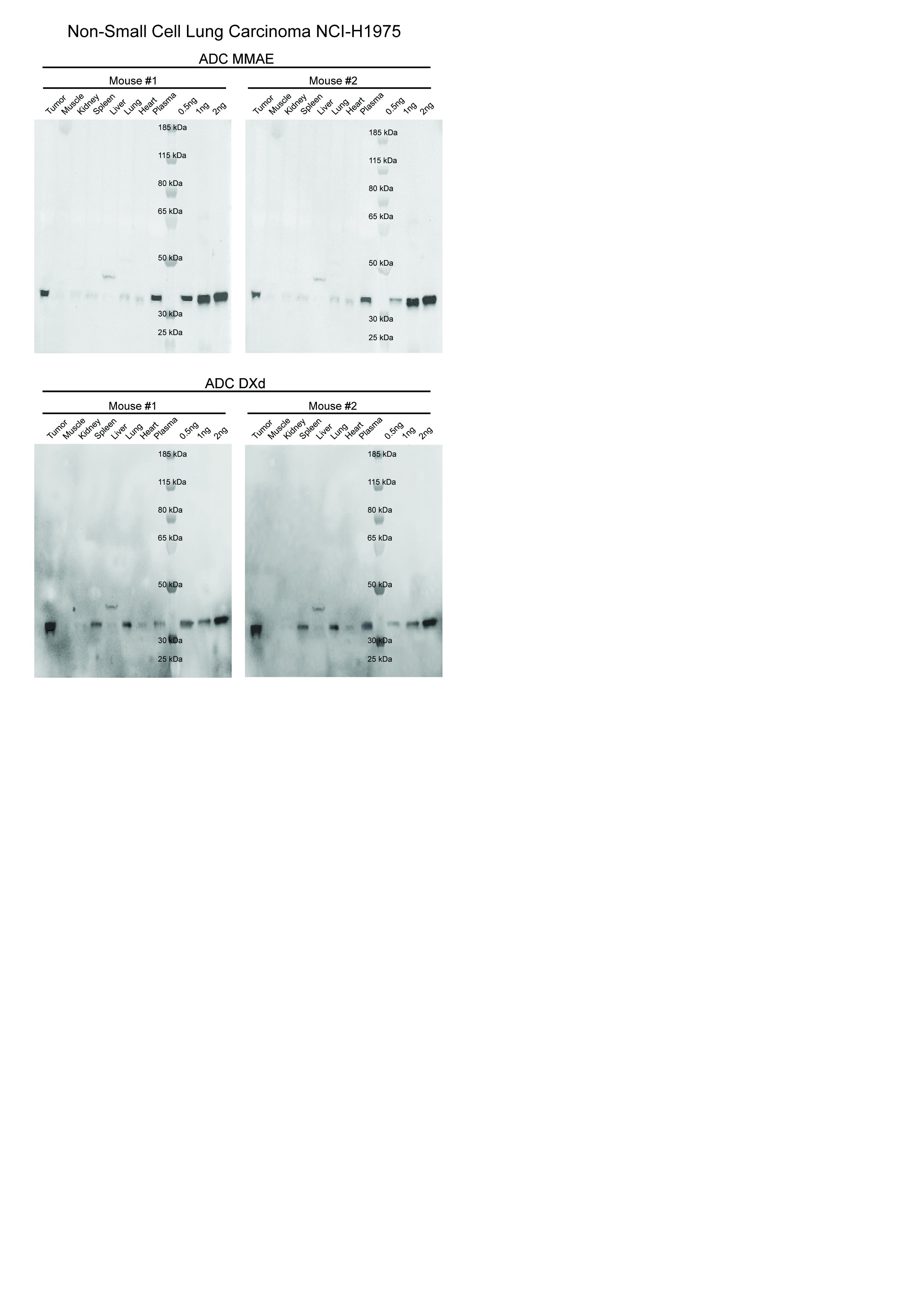

Supplement: Supplementary file 14 — Data Set 3 [file 41419_2026_8420_MOESM14_ESM.tif]
